# Discovery of a phase-separating small molecule that selectively sequesters tubulin in cells†

**DOI:** 10.1039/d1sc07151c

**Published:** 2022-04-20

**Authors:** Genyir Ado, Naotaka Noda, Hue T. Vu, Amelie Perron, Amarjyoti D. Mahapatra, Karla Pineda Arista, Hideaki Yoshimura, Daniel M. Packwood, Fumiyoshi Ishidate, Shin-ichi Sato, Takeaki Ozawa, Motonari Uesugi

**Affiliations:** Institute for Chemical Research, Kyoto University Uji Kyoto 611-0011 Japan uesugi@scl.kyoto-u.ac.jp; Graduate School of Medicine, Kyoto University Uji Kyoto 611-0011 Japan; Department of Chemistry, School of Science, The University of Tokyo Tokyo 113-0033 Japan; Institute for Integrated Cell-Material Sciences (WPI-iCeMS), Kyoto University Kyoto 606-8501 Japan; School of Pharmacy, Fudan University Shanghai 201203 China

## Abstract

Phase-separated membraneless organelles or biomolecular condensates play diverse functions in cells, however recapturing their characteristics using small organic molecules has been a challenge. In the present study, cell-lysate-based screening of 843 self-assembling small molecules led to the discovery of a simple organic molecule, named huezole, that forms liquid droplets to selectively sequester tubulin. Remarkably, this small molecule enters cultured human cells and prevents cell mitosis by forming tubulin-concentrating condensates in cells. The present study demonstrates the feasibility of producing a synthetic condensate out of non-peptidic small molecules for exogenous control of cellular processes. The modular structure of huezole provides a framework for designing a class of organelle-emulating small molecules.

## Abstract 2

A non-peptidic small molecule, *R*-huezole, phase separates to selectively sequester tubulin proteins to control the cell cycle. Its modular structure provides a framework for designing bioactive molecules to mimic membraneless organelles in cells.

## Graphical abstract

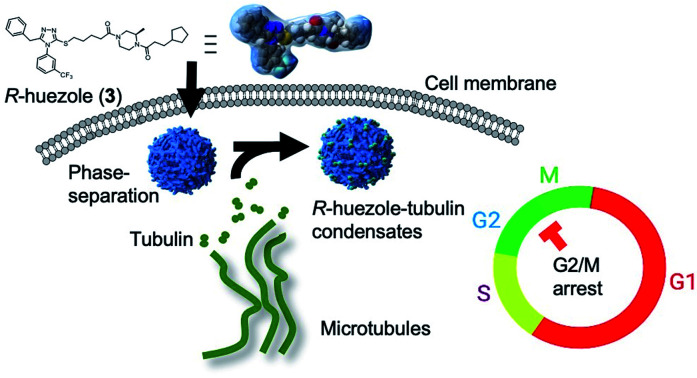

## Introduction

Compartmentalization is a defining characteristic of life. Cells organize their contents into organelles, which have classically been considered as membrane-separated architectures. However, a growing number of studies have revealed newly defined types of organelles that form *via* liquid–liquid phase separation, a physical process whereby components in a solution separate into two coexisting phases.^1,2^ These so-called membraneless organelles or biomolecular condensates can concentrate or sequester specific biomolecules and thereby potentiate or dampen specific biological reactions.

The phase-separated membraneless structures are associated with diverse cellular functions, including enzymatic reactions, degradation pathways, cellular homeostasis, and transcriptional control.^3–5^ One prominent role of membraneless organelles is the regulation of spindle formation during cell division at centrosomes and spindle formation sites.^6^ Centrosome is formed and expanded through phase separation at least in *C. elegans*,^7^ condensing tubulin to catalyze the generation of microtubules for assembly of the mitotic spindle.^8^ Spindle-defective protein 5 (SPD-5), one of the key centrosome proteins, forms phase-separated, spherical liquid condensates *in vitro*, which possess centrosomal activity for nucleating microtubules by recruiting tubulin and other centrosome proteins.^8^ A number of other spindle and centrosome-associated proteins, including BuGZ^9^ and TPX2,^10^ undergo liquid–liquid phase separation to recruit and concentrate tubulin for accelerating microtubule nucleation. Concentration of tubulin through liquid–liquid phase separation is a critical mechanism for regulating spindle formation during mitosis.

The widespread involvement of membraneless organelles in cellular functions has inspired the design of synthetic, artificial molecules that capture the characteristics of membraneless organelles.^11–14^ Such artificial organelle-like architectures would serve as models to further advance our understanding of the determinants of phase separation in cells and also open new opportunities for controlling biological processes. However, the artificial organelles that have been reported typically exploited biological components such as nucleic acids, proteins, and peptide building blocks.^15–18^ To date, only very limited success has been achieved using small molecules in formulating organelle-like structures that are capable of controlling biological processes.^19^ Creating artificial membraneless organelles with small organic molecules would offer simple models and alternatives for recreating or controlling cellular events and also enable unique opportunities for future drug discovery by interfering with disease processes. In the present study, we have taken a screening approach to search for small organic molecules that form artificial organelle-like condensates.

## Results and discussion

### Discovery of huezole

**Fig. 1:**
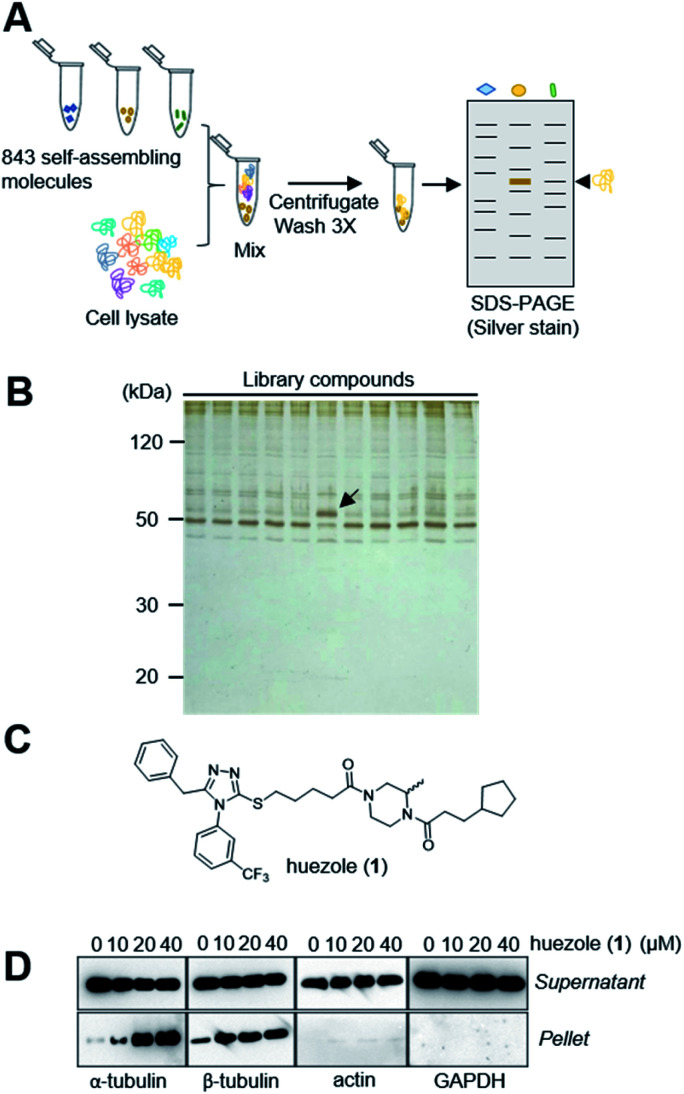
Discovery of huezole. (A) A schematic diagram of the screening procedure. (B) A representative silver-stained image of a screening gel of self-assembly library compounds. The protein band specific for huezole (1) is marked with an arrow. (C) Chemical structure of huezole. (D) Dose-dependent co-precipitation of tubulin with huezole. Immunoblots of supernatant and pellet fractions with indicated antibodies are shown.

Naturally occurring membraneless organelles often concentrate or sequester specific proteins. In order to search for phase-separating small molecules that specifically interact with cellular proteins, we exploited our previously reported chemical library of self-assembling small molecules.^20^ Each of the compounds in our library form particles detectable by one of two environment-sensitive fluorescent probes, namely Nile Red and 8-anilinonaphthalene-1-sulfonic acid (ANS), in PBS buffer, and display a variety of particle morphologies such as micelle, fibers, crystals, and colloid aggregates.^20^ Each of the library compounds (20 μM) was incubated with human cell lysates. After this incubation period, the lysates were subjected to centrifugation (Fig. 1A). The proteins which co-precipitated with small-molecule assemblies were analysed by SDS-PAGE. Over 95% of the 843 self-assembling molecules displayed similar patterns of co-precipitated proteins, which either non-specifically interacted with the assembled materials or denatured during the experiments (Fig. 1B). The screening led to the discovery of the simple molecule which we named huezole (1) (Fig. 1C). This molecule displayed the most selective co-sedimentation with a particular protein (Fig. 1B). Repeated experiments showed that the degree of the co-precipitation with the ∼50 kDa protein is dependent on the concentrations of huezole (1) (Fig. S1†). Excision of the ∼50 kDa band, followed by in-gel digestion and LC-MS analysis, unambiguously established the co-precipitated protein as tubulin, a cytoskeletal heterodimer protein that consists of α and β subunits. Western blot analysis of the co-precipitated proteins showed that huezole co-precipitates with alpha, beta and gamma subunits of tubulin but not with actin and GAPDH (Fig. 1D and S2†). Overall, these experiments indicate that tubulin subunits associate selectively with the huezole assembly.

**Fig. 2:**
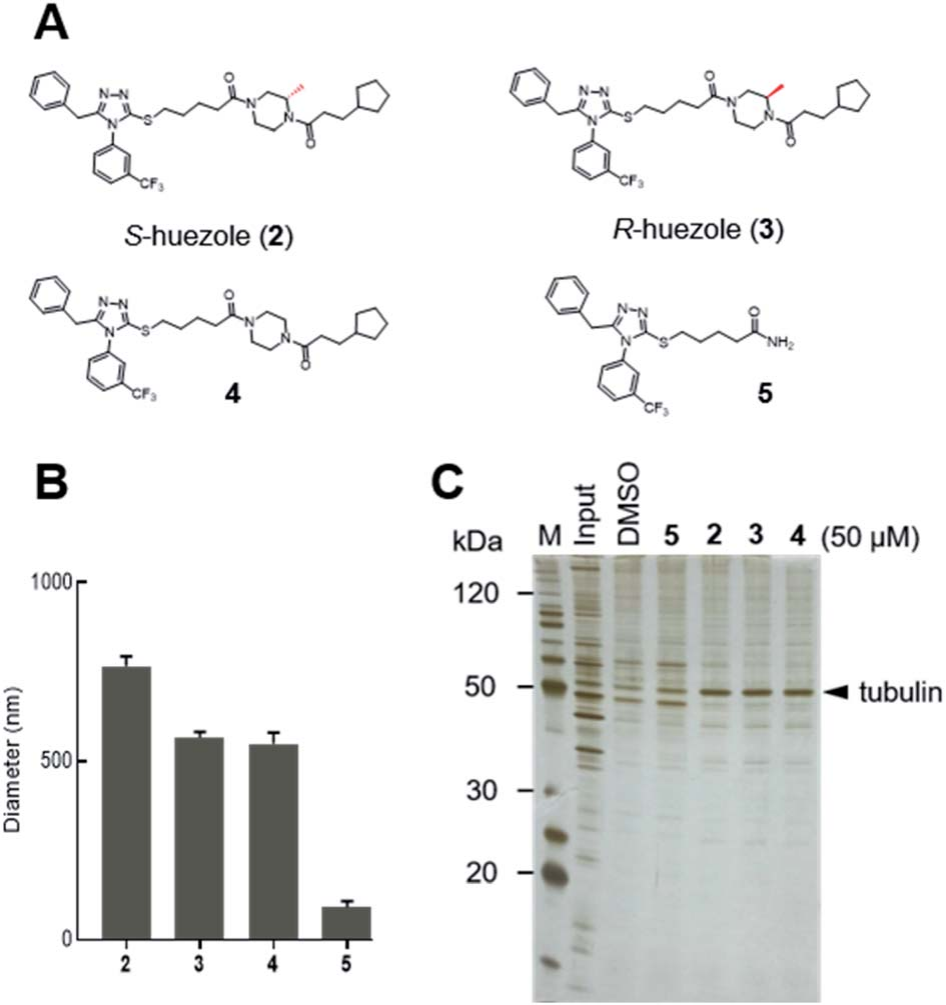
Self-assembling properties of huezole analogues 2–4. (A) Chemical structures of huezole analogues 2–5. (B) Average hydrodynamic diameters of the compounds at 50 μM in PBS. (C) Silver-stained gel image showing the co-precipitation of tubulin with analogues 2–5 in cell lysates.

Huezole (1), as stored in the library, is a racemic mixture. To examine the importance of its stereochemistry, we synthesized its *S* and *R* isomers (2 and 3, respectively) and an achiral analogue lacking the methyl group (4) (Fig. 2A). Dynamic light scattering (DLS) measurements indicate that molecules 2, 3, and 4 all form particles with 500–800 nm diameters (Fig. 2B). Their assemblies co-precipitated with tubulin equally well at 50 μM in cell lysates (Fig. 2C), suggesting that the stereochemistry at the piperazine group is dispensable for self-assembly and tubulin interaction of huezole. In contrast, removal of the right-hand half (molecule 5) eliminated the ability to self-assemble and co-precipitate with tubulin (Fig. 2B, C, and S3†), indicating that the piperazine amide moiety is required for the activity. Molecule 5 serves as a negative control for later studies.

### Analysis of huezole-tubulin condensates

**Fig. 3:**
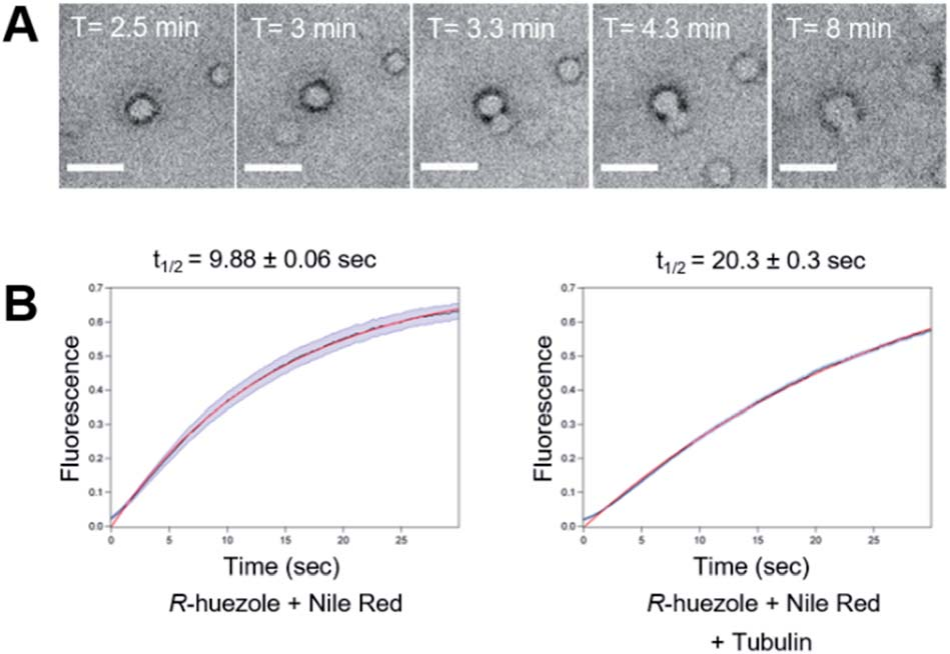
Liquid-like phase separation of *R*-huezole (3). (A) Bright field time-lapse images of coalescing puncta of *R*-huezole (3). Scale bars, 5 μm. (B) FRAP assays of Nile-Red-doped *R*-huezole particles (90 nM Nile Red and 50 μM *R*-huezole). Recovery of the normalized fluorescence intensities indicated *t*_1/2_ values in the absence (9.88 s) and presence (20.3 s) of tubulin (200 nM). The blue lines indicate SEM, and the red lines show the results of exponential fitting with the average intensities of fluorescent particles (*N* = 30 for Nile Red + *R*-huezole and *N* = 94 for Nile Red + *R*-huezole + tubulin, respectively).

We focused on *R*-huezole (3) for further in-depth analysis. The sub-micron size of the *R*-huezole particles allowed us to monitor their properties under a confocal microscope. The huezole assemblies were visualized by Nile Red (100 nM), a typical environment-sensitive fluorescent probe for molecular condensates.^20,21^*R*-huezole demixed in an aqueous solution and formed liquid-like spherical droplets, which can fuse with one another to generate larger spherical units *in vitro* (Fig. 3A). In PBS buffer, the formation of droplets at 25 μM took 10 min to complete (Fig. S4†). Such a slow saturation following an initial fast demixing has been reported for biomolecular condensates.^11^ To quantify the mobility of molecules inside droplets, we conducted fluorescence recovery after photobleaching (FRAP) experiments, in which the center of a Nile Red-doped droplet of *R*-huezole was photo-bleached with high-intensity laser light in a total internal reflection fluorescence microscope, with the fluorescence recovery of the photobleached area was followed thereafter. The half-maximal recovery time (*t*_1/2_) of the fluorescence was 9.8 ± 0.06 s (Fig. 3B), demonstrating that Nile Red molecules are mobile within *R*-huezole assemblies and exchange rapidly with the surroundings. In the presence of tubulin, the recovery rate increased (*t*_1/2_ = 20.3 ± 0.3 s) but remained comparable to those reported for phase-separated fluidic liquid droplets of proteins.^2,8,22^ Collectively, these results indicate that *R*-huezole gradually forms phase-separated liquid-like droplets in an aqueous solution.

**Fig. 4:**
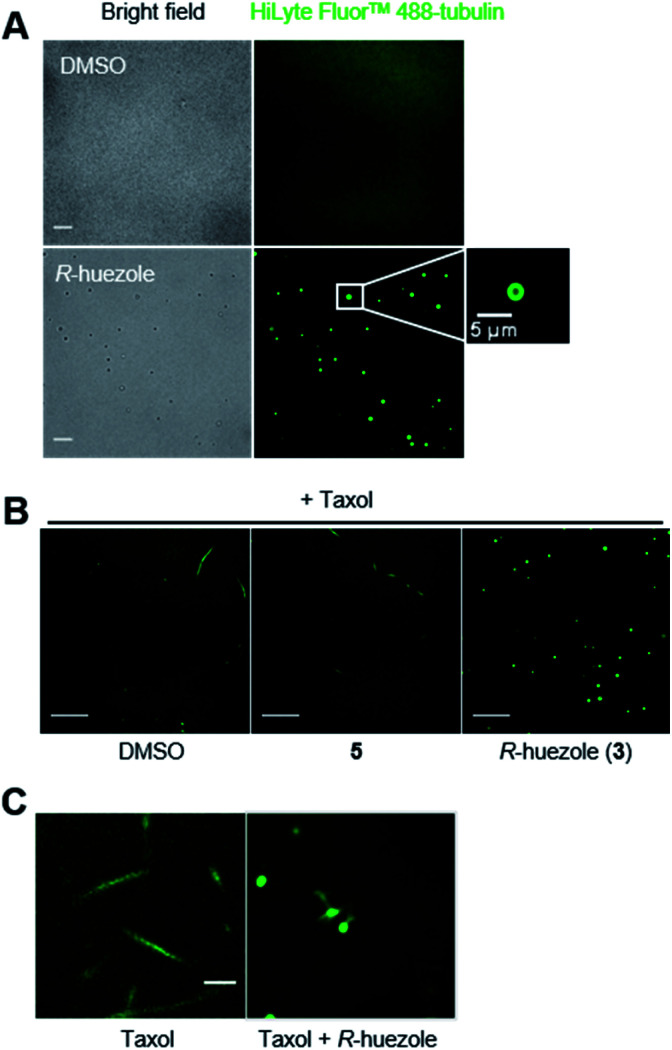
Microscopic observation of the effects of *R*-huezole (3) on tubulin *in vitro*. (A) *In vitro* tubulin sequestration with *R*-huezole (3). Labeled tubulin (200 nM) was incubated with DMSO (1%) or *R*-huezole (50 μM) for 30 min before imaging. Scale bars, 10 μm. (B) Effects of *R*-huezole (3) on Taxol-induced tubulin polymerization *in vitro*. Labeled tubulin was dissolved in G-PEM buffer including Taxol (1 μM) followed by incubation with 50 μM *R*-huezole (3) for 30 min at 37 °C prior to imaging. Scale bars, 20 μm. (C) *R*-huezole-tubulin condensates under a highly polymerization-inducing condition. Polymerization of labeled tubulin (900 nM) was induced overnight by Taxol (50 μM) in the presence or absence of 50 μM *R*-huezole (3) in PBS. Scale bar, 5 μm.

To observe the interaction of the huezole droplets with tubulin *in vitro*, HiLyte Fluor™ 488-labeled tubulin was incubated with *R*-huezole in PBS buffer. Confocal imaging indicated that tubulin was sequestered within the *R*-huezole droplets (Fig. 4A). The interaction was competed off with an excess amount of non-labeled tubulin but not with actin, consistent with the idea that the interaction is selective (Fig. S5†).

Tubulin is capable of polymerizing *in vitro* to form microtubules in a G-PEM buffer (80 mM PIPES, pH 6.9, 2 mM MgCl_2_, 1 mM GTP, and 0.5 mM EGTA) containing Taxol, a microtubule stabilizer. In fact, under such a polymerization-enabling condition, we were able to observe the microtubule fiber formation of HiLyte Fluor™ 488-labeled tubulin under a confocal microscope (Fig. 4B). *R*-huezole trapped HiLyte Fluor™ 488-labeled tubulin into its droplets to abrogate the microtubule formation, while no tubulin droplets were observed in the presence of molecule 5, which was the negative control (Fig. 4B). Of note, the treatment with molecule 5 led to slight shortening of the microtubules (Fig. S6†).

To gain insights into their effects on polymerization speed, we employed a kinetic mode, absorbance-based tubulin polymerization assay.^23,24^*R*-huezole (3) inhibited tubulin polymerization more potently than molecule 5 did (Fig. S7†). However, the polymerization was not completely blocked by *R*-huezole (3), suggesting that *R*-huezole is capable of ectopically squelching tubulin but not fully disabling its polymerization. In fact, extended incubation of the *R*-huezole-tubulin condensates under a highly polymerization-inducing condition nucleated microtubules to form a centrosome-like microtubule aster similar to those previously observed *in vitro* with spindle-forming protein condensates^8,25^ (Fig. 4C).

### Cellular activities of huezole

**Fig. 5:**
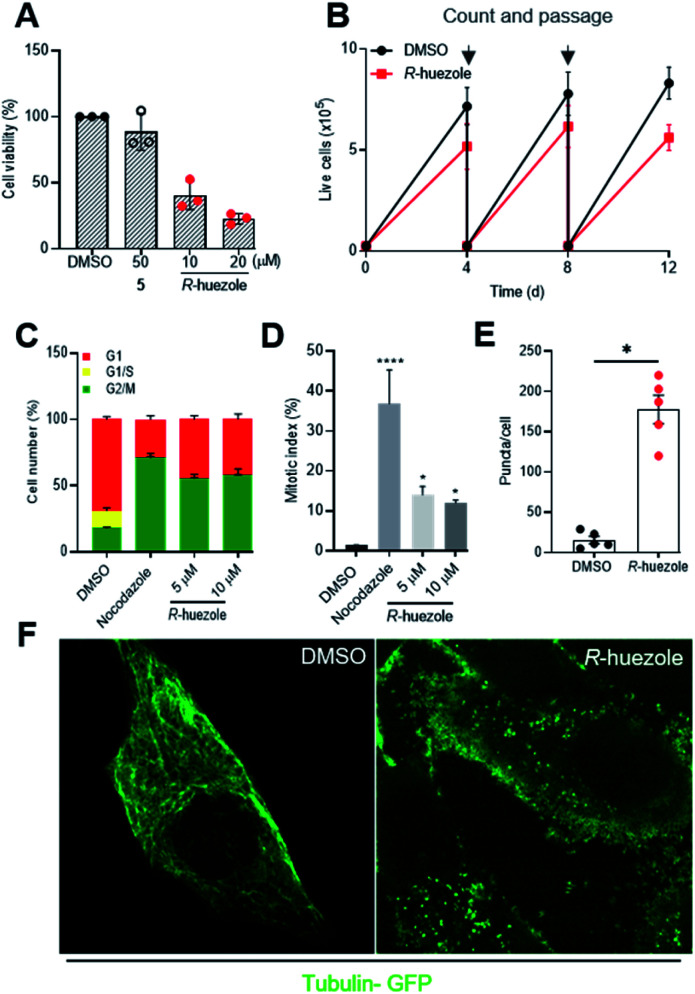
Effects of *R*-huezole (3) in cells. (A) HeLa cells were treated with DMSO (0.5%), 5 (50 μM), or *R*-huezole (10 and 20 μM). Cell viability was monitored by WST-8 assay after 48 h. (B) *R*-huezole (3) delays the proliferation of HeLa cells. Passage-based cell proliferation was monitored at 4, 8, or 12 days after treatment with DMSO or *R*-huezole (5 μM). (C) *R*-huezole (3) treatment leads to G2/M cell-cycle arrest. HEK293 cells were transfected with Fucci fluorescent probes mCherry-hCdt1 (30/120) (red) and AmCyan-hGeminin (green) followed by treatment with DMSO or *R*-huezole (3). An increase in AmCyan-hGeminin (green) fluorescence indicates an increase in G2/M cell population. Nocodazole (1 μM) was used as a positive control. (D) Effects of *R*-huezole (3) on mitotic index (% of cells with condensed chromatin). HEK293 cells were stained for nucleus with Hoechst 33342 after 48 h treatment with *R*-huezole. Data represent average values mean ± S.D (*n* = 3). The *p* values (*p < 0.05 and ****p < 0.0001) were determined using one-way ANOVA followed by Dunnett's multiple comparisons test with a 95% confidence interval. (E) Cellular tubulin sequestration with *R*-huezole (3). HeLa cells were transduced with CellLight™ tubulin-GFP followed by treatment with DMSO or *R*-huezole (50 μM) for 3 h prior to imaging. HeLa cells were analysed for puncta and 5 cells from at least 7 microscopic fields were quantified with similar results from three independent experiments. Data represent average values mean ± S.D (*n* = 3). The *p* value (*p < 0.05) was determined by an unpaired two-tailed Student's *t*-test. (F) Representative confocal images of the 3-induced cellular tubulin sequestration. DMSO-treated cells (left) show uniform tubulin-GFP expression; *R*-huezole treated cells (right) show intracellular puncta indicating sequestration with *R*-huezole.

Tubulin is a building block for microtubules, a protein assembly that drives cell division and intracellular transport.^26–28^ Cellular sequestration of tubulin by *R*-huezole may alter the growth of cultured human cells. Exposure of HeLa cells to *R*-huezole for 2 days reduced the cell growth in a dose-dependent manner with an IC_50_ value of 4.4 μM (Fig. S8†), whereas molecule 5 had little effect even at 50 μM (Fig. 5A). The anti-proliferation activity was confirmed by a passage-based proliferation assay,^29^ in which we treated HeLa cells with 5 μM of *R*-huezole and routinely counted, passaged, and re-seeded the cells for 12 days. The results highlighted the ability of *R*-huezole to retard cell proliferation over time (Fig. 5B).

In cells undergoing cell division, the organized polymerization of tubulin to microtubules at centrosomes allows formation of spindle fibers and polarized migration of duplicated chromosomes during mitosis. The anti-proliferation activity of *R*-huezole and the major role of tubulin in mitosis prompted us to test the effects of *R*-huezole on the cell cycle. HEK293 cells were transfected with the fluorescent ubiquitination-based cell-cycle indicators (Fucci).^30^ These probes exploit the cell cycle-dependent proteolysis of Cdt1 and geminin by E3 ligases, enabling the visualization of G1 and G2/M phases in red and green, respectively. Treatment with *R*-huezole induced a dose-dependent increase in the geminin/Cdt1 fluorescence ratio, suggesting an increase in the cell population in G2/M (Fig. 5C and S9A†). A similar increase was also observed with the microtubule-depolarizing agent nocodazole, which is known to arrest cell cycle progression in G2/M (ref. 31) (Fig. 5C and S9A†). As the Fucci probes are unable to distinguish between G2 and M phases of the cell cycle, we examined the effect of *R*-huezole on chromatin condensation in order to evaluate the percentage of cells undergoing mitosis as shown in (Fig. 5D). It was found that *R*-huezole increased the proportion of cells with visible chromosomes from 1.5 to 14%. Fluorescence microscopy analysis also showed incomplete chromosome congression at the metaphase plate, suggesting that the cells are blocked in the mitotic prometaphase (Fig. S9B†).

The potent cellular activity of *R*-huezole led us to investigate whether *R*-huezole forms condensates with tubulin in cells as observed *in vitro*. When we preincubated *R*-huezole in the culture medium for 5 min before the exposure to the cells, the anti-proliferation activity of *R*-huezole was compromised (Fig. S10†), indicating that the extracellular droplet formation of *R*-huezole limited its availability within the cells. Considering the slow assembling rate of *R*-huezole, it is likely that non-assembled, diffused molecules of *R*-huezole penetrate the cell membrane to undergo intracellular formation of condensates. To detect these intracellular condensates, we treated HeLa cells expressing tubulin-GFP with *R*-huezole. Using confocal microscopy, we observed the formation of submicron-scale punctate assemblies of tubulin-GFP in the presence of *R*-huezole (Fig. 5E and F). The average number of puncta per cell was estimated to be 172 (Fig. 5E). Unlike centrosomes, the huezole droplets were unable to nucleate spindle fibers in the cells due to the inability to recruit other centrosome proteins required for the spindle fiber formation. For example, huezole failed to display apparent co-precipitation *in vitro* with pericentrin, a centrosome protein involved in the spindle formation (Fig. S2†). Although we observed only a limited number of cells undergoing metaphase, close inspection of those mitotic cells suggests that the *R*-huezole-induced puncta of tubulin led to the distortion of the spindle fibers (Fig. S11†). These results collectively suggest that *R*-huezole prevents cell mitosis by forming a large number of tubulin-concentrating, phase-separated condensates in cells.

We examined whether 1,6-hexanediol, a widely used reagent to dissolve liquid–liquid phase separated condensates, disrupts the huezole assemblies. Particle formation of *R*-huezole was monitored by DLS in the presence of 1,6-hexanediol. Increasing concentrations of 1,6-hexanediol decreased the number of *R*-huezole particles (Fig. S12A†). Confocal microscopic observation of *R*-huezole-sequestered labeled tubulin indicated that 1,6-hexanediol dissolves the *R*-huezole-tubulin assemblies *in vitro* (Fig. S12B and C†). Effects of 1,6-hexanediol on the *R*-huezole-tubulin assemblies were also examined in HeLa cells. Addition of 5% 1,6-hexanediol decreased the fluorescent intensity of the *R*-huezole-induced puncta of tubulin-GFP in cells (Fig. S12D and E†). Due to the cytotoxicity of 1,6-hexanediol in HeLa cells (Fig. S12F†), it was challenging to accurately evaluate its effects on the anti-proliferation activity of *R*-huezole in live cells.^32^ Nevertheless, normalized cell viability data suggested that 1,6-hexanediol mitigated the anti-proliferation activity of *R*-huezole (Fig. S12G†). Overall these results support the notion that *R*-huezole forms liquid-like tubulin condensates in cells.

### Modular structure of huezole

Our study indicates that the intracellularly formed condensates of *R*-huezole sequester tubulin from centrosomes or spindle formation sites to block cell mitosis. This is reminiscent of naturally occurring biomolecular condensates that inhibit protein functions by sequestering molecules from their sites of action. In this regard, a number of synthetic approaches have been developed to control biological events by sequestering biomolecules to defined locations. Examples include the use of coacervates,^33–36^ hydrogels,^37–39^ micellar assemblies,^40^ porous materials^41^ as well as peptide assemblies.^42–44^ Although the huezole condensates are not perfect mimetics of centrosome, huezole may represent the small organic molecules that exert biological activity by capturing characteristics of cellular biomolecular condensates.

In the structure of huezole, we observe two features that mimic naturally occurring proteins in membraneless organelles. Namely a flexible tail for condensation and a more ordered structure for association with other proteins. Our structure–activity relationship study described above (Fig. 2 and 5) showed that the piperazine amide tail of huezole is required for self-assembly. To gain additional insights into the role of the piperazine amide tail, we conducted solution-sate NMR spectroscopy experiments, in which we used an achiral version of huezole (molecule 4) to simplify NMR signals. Given that molecule 4 is completely soluble in DMSO but forms assemblies in H_2_O, NMR studies in a mixture of D_2_O and DMSO-*d*_6_ may permit detection of an intermediate state to locate condensation-prone segments of the molecule. Molecule 4 was dissolved in DMSO-*d*_6_ in the presence of varying amounts of D_2_O, and NOESY spectra of the samples were recorded. Although molecule 4 in 100% DMSO-*d*_6_ exhibited essentially no negative NOE signals, increasing concentrations of D_2_O from 0 to 10% resulted in the appearance of clear negative NOE crosspeaks among protons in the piperazine amide tail (Fig. S13†). The results suggest that this flexible segment has the propensity to self-assemble in an aqueous solution, most likely driving the droplet formation. On the other hand, no detectable NOEs appeared for the benzylphenyl triazole moiety in the presence of 10% D_2_O, suggesting that the more rigid aromatic segment plays little or no role in driving the droplet formation.

The importance of the piperazine amide tail was further supported by molecular dynamics simulation, in which two molecules of 4 in water were simulated for 10 ns. Simulation details can be found in the ESI.† The simulation showed no detectable hydrogen bonding or coordination between the water solvents and the molecules, suggesting poor solvation (Fig. S14A†). The weak association of the two molecules were mediated primarily by van der Waals (vdW) interactions between phenyl and piperazine amide moieties (Fig. S14B–D†). These interactions are hydrophobic in character and exclude water molecules from the region between molecules. This exclusion of water molecules is a necessary condition for phase separation in aqueous solvent, and support the role of the piperazine amide tail in forming liquid-like droplets.

We considered the possibility that the benzylphenyl triazole moiety engages in the interaction with tubulin, as aromatic group-rich compounds tend to bind to tubulin or microtubules. To test the hypothesis, we synthesized a fluorescent probe (probe 6) in which the piperazine amide tail of huezole was replaced by TAMRA, a red-fluorescent small-molecule tag. The probe (0.1 μM) was incubated with increasing concentrations of tubulin, and the fluorescence polarization was measured. Tubulin increased fluorescence of the probe but not that of free TAMRA, indicating that the benzylphenyl triazole moiety interacts with tubulin (Fig. S15†). The dissociation constant (*K*_D_) of the interaction was estimated to be 3.5 μM, comparable to the IC_50_ value of 4.4 μM (Fig. S8†) for the anti-proliferation activity of *R*-huezole. In line with this result, addition of increasing concentrations of the benzylphenyl triazole moiety alone (molecule 5) had little impacts on the particle diameters of 1 and 3 as measured by DLS (Fig. S16A†).

Given that molecule 5 has weak affinity to tubulin, it could influence the tubulin binding of 1 and 3. However, co-precipitation assays with increasing concentrations (up to 4 times excess) of 5 exhibited limited effects on the tubulin co-precipitation with 1 and 3 (Fig. S16B†). Confocal microscopic observation of labeled tubulin also indicated that addition of molecule 5 had no detectable effects on the tubulin sequestration with molecule 3 (Fig. S17A and B†). These results suggest a role of multivalency of the huezole assembly in enhancing its interaction with tubulin and in forming tubulin-condensing assemblies.

Consistent with the role of the benzylphenyl triazole moiety in tubulin binding, both molecules 3 and 5 displayed inhibition of tubulin polymerization, although molecule 3 did so more potently than molecule 5 (Fig. S7†). Given that molecule 5 has no detectable effects on cell viability, the consequence of *R*-huezole (3) on cell viability is unlikely to stem solely from an impact on microtubule polymerization. Intracellular formation of tubulin-condensing assemblies could also influence cell viability through microtubule-independent mechanisms.

## Conclusions

The present study demonstrates the feasibility of producing a synthetic condensate out of a non-peptidic small molecule for exogenous control of cellular processes. The modular structure of huezole presents an excellent framework for designing a class of bioactive self-assembling small molecules that possess two key features, a covalent conjugation of a protein ligand and a self-assembling unit. Such self-assembling small molecules, with their cell permeability, stability, and chemical tractability, may open new possibilities for organelle-emulating molecules that complement the more established peptide-or protein-based artificial organelles.

## Data availability

The authors declare that all data supporting the findings of this study are available within the article and ESI.†

## Author contributions

MU conceptualised and supervised the project. GA performed the research. NN and ADM performed the synthesis. HY performed the FRAP experiment. DMP performed the MD simulation. FI and GA performed the Airyscan LSM-880 confocal imaging. GA, NN, HTV, AP, KPA, HY, DMP, FI, SS and MU contributed to the analysis and discussions during the work. GA and MU prepared the manuscript with edits from all co-authors. TO and MU obtained funding for this work. All authors have approved the final version of the manuscript.

## Conflicts of interest

There are no conflicts to declare.

## Supplementary Material

SC-013-D1SC07151C-s001

## References

[cit1] Shin Y., Brangwynne C. P. (2017). Science.

[cit2] Banani S. F., Lee H. O., Hyman A. A., Rosen M. K. (2017). Nat. Rev. Mol. Cell Biol..

[cit3] O'Flynn B. G., Mittag T. (2021). Curr. Opin. Cell Biol..

[cit4] Hnisz D., Shrinivas K., Young R. A., Chakraborty A. K., Sharp P. A. (2017). Cell.

[cit5] Ditlev J. A., Case L. B., Rosen M. K. (2018). J. Mol. Biol..

[cit6] Ong J. Y., Torres J. Z. (2020). Mol. Cell.

[cit7] Raff J. W. (2019). Trends Cell Biol..

[cit8] Woodruff J. B., Gomes B. F., Widlund P. O., Mahamid J., Honigmann A., Hyman A. A. (2017). Cell.

[cit9] Jiang H., Wang S., Huang Y., He X., Cui H., Zhu X., Zheng Y. (2015). Cell.

[cit10] King M. R., Petry S. (2020). Nat. Commun..

[cit11] Li P., Banjade S., Cheng H. C., Kim S., Chen B., Guo L., Llaguno M., Hollingsworth J. V., King D. S., Banani S. F., Russo P. S., Jiang Q. X., Nixon B. T., Rosen M. K. (2012). Nature.

[cit12] Reinkemeier C. D., Girona G. E., Lemke E. A. (2019). Science.

[cit13] Heidenreich M., Georgeson J. M., Locatelli E., Rovigatti L., Nandi S. K., Steinberg A., Nadav Y., Shimoni E., Safran S. A., Doye J. P. K., Levy E. D. (2020). Nat. Chem. Biol..

[cit14] Dzuricky M., Rogers B. A., Shahid A., Cremer P. S., Chilkoti A. (2020). Nat. Chem..

[cit15] Pieszka M., Han S., Volkmann C., Graf R., Lieberwirth I., Landfester K., Ng D. Y. W., Weil T. (2020). J. Am. Chem. Soc..

[cit16] Nott T. J., Craggs T. D., Baldwin A. J. (2016). Nat. Chem..

[cit17] Guo X., Li F., Liu C., Zhu Y., Xiao N., Gu Z., Luo D., Jiang J., Yang D. (2020). Angew. Chem., Int. Ed..

[cit18] Schuster B. S., Reed E. H., Parthasarathy R., Jahnke C. N., Caldwell R. M., Bermudez J. G., Ramage H., Good M. C., Hammer D. A. (2018). Nat. Commun..

[cit19] Kato M., Han T. W., Xie S., Shi K., Du X., Wu L. C., Mirzaei H., Goldsmith E. J., Longgood J., Pei J., Grishin N. V., Frantz D. E., Schneider J. W., Chen S., Li L., Sawaya M. R., Eisenberg D., Tycko R., McKnight S. L. (2012). Cell.

[cit20] Jin S., Vu H. T., Hioki K., Noda N., Yoshida H., Shimane T., Ishizuka S., Takashima I., Mizuhata Y., Pe K. B., Ogawa T., Nishimura N., Packwood D., Tokitoh N., Kurata H., Yamasaki S., Ishii K. J., Uesugi M. (2021). Angew. Chem., Int. Ed..

[cit21] Hawe A., Sutter M., Jiskoot W. (2008). Pharm. Res..

[cit22] Nosella M. L., Tereshchenko M., Pritišanac I., Chong P. A., Toretsky J. A., Lee H. O., Forman-Kay J. D. (2021). J. Am. Chem. Soc..

[cit23] Shelanski M. L., Gaskin F., Cantor C. R. (1973). Proc. Natl. Acad. Sci. U. S. A..

[cit24] Lee J. C., Timasheff S. N. (1977). Biochemistry.

[cit25] Hernández-Vega A., Braun M., Scharrel L., Jahnel M., Wegmann S., Hyman B. T., Alberti S., Diez S., Hyman A. A. (2017). Cell Rep..

[cit26] Nogales E., Wolf S. G., Downing K. H. (1998). Nature.

[cit27] Meunier S., Vernos I. (2012). J. Cell Sci..

[cit28] Rizzelli F., Malabarba M. G., Sigismund S., Mapelli M. (2020). Open Biol..

[cit29] Meitinger F., Ohta M., Lee K. Y., Watanabe S., Davis R. L., Anzola J. V., Kabeche R., Jenkins D. A., Shiau A. K., Desai A., Oegema K. (2020). Nature.

[cit30] Choi H. J., Fukui M., Zhu B. T. (2011). PLoS One.

[cit31] Vasquez R. J., Howell B., Yvon A. M., Wadsworth P., Cassimeris L. (1997). Mol. Biol. Cell.

[cit32] Düster R., Kaltheuner I. H., Schmitz M., Geyer M. (2021). J. Biol. Chem..

[cit33] Deshpande S., Brandenburg F., Lau A., Last M. G. F., Spoelstra W. K., Reese L., Wunnava S., Dogterom M., Dekker C. (2019). Nat. Commun..

[cit34] Sokolova E., Spruijt E., Hansen M. M. K., Dubuc E., Groen J., Chokkalingam V., Piruska A., Heus H. A., Huck W. T. S. (2013). Proc. Natl. Acad. Sci. U. S. A..

[cit35] Tang T. Y. D., Hak C. R. C., Thompson A. J., Kuimova M. K., Williams D. S., Perriman A. W., Mann S. (2014). Nat. Chem..

[cit36] Nakashima K. K., Vibhute M. A., Spruijt E. (2019). Front. Mol. Biosci..

[cit37] Battig M. R., Huang Y., Chen N., Wang Y. (2014). Biomaterials.

[cit38] Vulic K., Shoichet M. S. (2012). J. Am. Chem. Soc..

[cit39] Aufinger L., Simmel F. C. (2018). Angew. Chem., Int. Ed..

[cit40] Maity D., Howarth M., Vogel M. C., Magzoub M., Hamilton A. D. (2021). J. Am. Chem. Soc..

[cit41] Bhattacharya A., Niederholtmeyer H., Podolsky K. A., Bhattacharya R., Song J. J., Brea R. J., Tsai C. H., Sinha S. K., Devaraj N. K. (2020). Proc. Natl. Acad. Sci. U. S. A..

[cit42] Feng Z., Wang H., Xu B. (2018). J. Am. Chem. Soc..

[cit43] Wang H., Feng Z., Xu B. (2019). J. Am. Chem. Soc..

[cit44] He H., Liu S., Wu D., Xu B. (2020). Angew. Chem., Int. Ed..

