# Supplementary material for: Discovery of a phase-separating small molecule that selectively sequesters tubulin in cells
Source: Chem Sci. 2022 Apr 20;13(19):5760–6. doi: 10.1039/d1sc07151c (PMC9116451; doi:10.1039/d1sc07151c)
Supplement: SC-013-D1SC07151C-s001 [file SC-013-D1SC07151C-s001.pdf]

Electronic Supplementary Material (ESI) for Chemical Science.
This journal is © The Royal Society of Chemistry 2022

Supporting Information

Discovery of a Phase-Separating Small Molecule That Selectively Sequesters Tubulin in Cells

Genyir Ado,[ab] Naotaka Noda,[ab] Hue T. Vu,[ab] Amelie Perron,[ad] Amarjyoti D. Mahapatra,[a] Karla Pineda Arista,[ab] Hideaki Yoshimura,[c] Daniel M. Packwood,[d] Fumiyoshi Ishidate,[d] Shin-ichi Sato,[a] Takeaki Ozawa,[c] and Motonari Uesugi*[ade]

[a] *Institute for Chemical Research, Kyoto University, Uji, Kyoto 611-0011, Japan*
[b] *Graduate School of Medicine, Kyoto University, Uji, Kyoto 611-0011, Japan*
[c] *Department of Chemistry, School of Science, The University of Tokyo, Tokyo 113-0033, Japan*
[d] *Institute for Integrated Cell-Material Sciences (WPI-iCeMS), Kyoto University, Kyoto 606-8501, Japan*
[e] *School of Pharmacy, Fudan University, Shanghai 201203, China*

Table of Contents

Supplemental Figures ……………………………………………………………..02

Chemical Synthesis …..………………………………………………………………19

Experimental Procedures ……………………………………………………………25

Compounds NMR Data ………………….……………………………………………..29

References ………….……………………………………………………….…….……35

Supplemental Figures

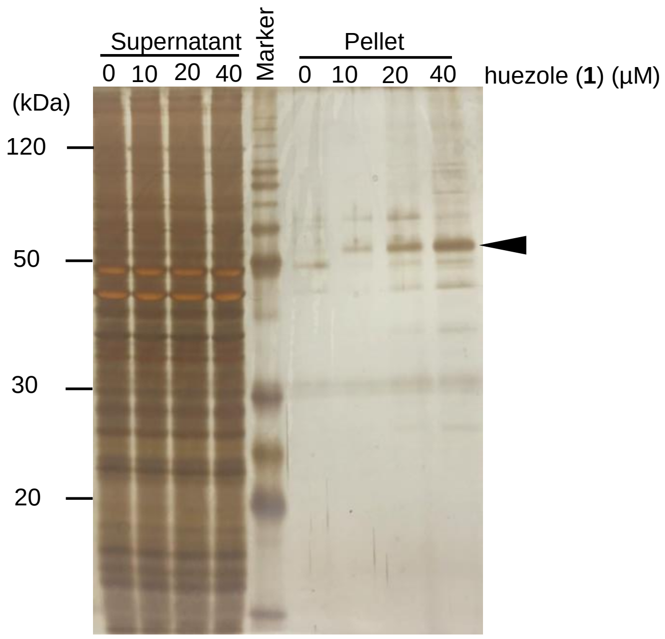

Figure S1. Dose dependent co-precipitation of the ~50 kDa protein with huezole. HeLa cell lysates (50 μL) were treated with DMSO or *R*-huezole (10, 20 or 40 μM) and incubated for 30 min at 4°C. The samples were centrifuged (12000 rpm, 10 min, 4°C), and then washed with PBS buffer (3x). The supernatant and pellet protein fractions were resolved on a 12% SDS-PAGE and analyzed by silver staining. The band of the ~50 kDa protein is indicated by an arrowhead.

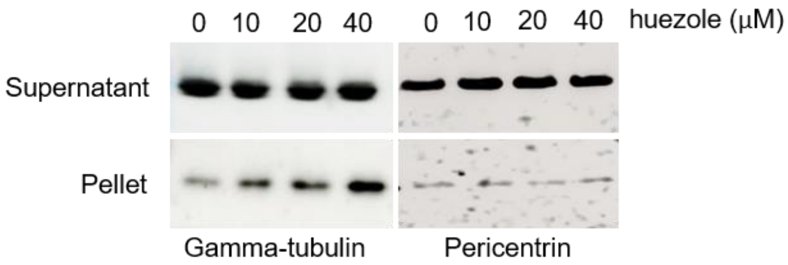

Figure S2. Immunoblot image representing dose dependent co-precipitation of the gamma-tubulin with huezole. HeLa cell lysates (50 µL) were treated with DMSO or huezole (10, 20 or 40 µM) and incubated for 30 min at 4°C. The samples were centrifuged (12000 rpm, 10 min, 4°C), and then washed with PBS buffer (3x). The supernatant and pellet protein fractions were resolved on a 12% SDS-PAGE and analyzed by silver staining. Centrosomal protein pericentrin does not display apparent co-precipitation with huezole suggesting selective interaction with tubulin subtypes.

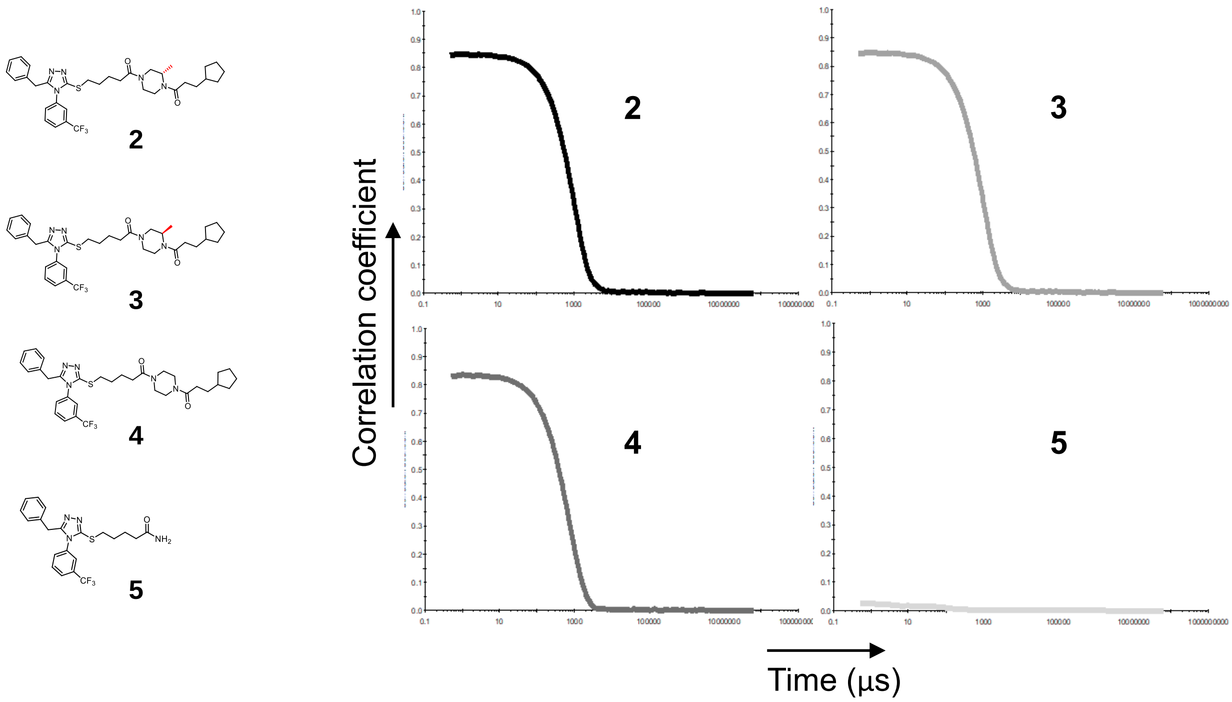

Figure S3. Correlation coefficient functions of synthesized molecules (**2**, **3**, **4**, and **5**) as measured by DLS (Zetasizer Nano-S). The calculated diameters of particles are shown in Figure 2B.

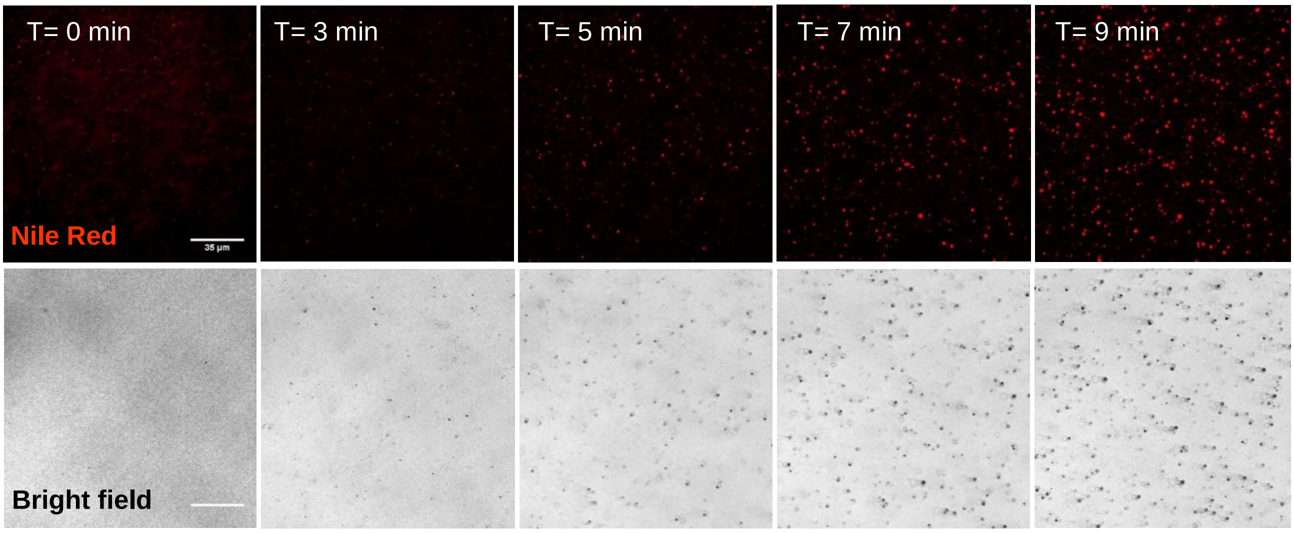

Figure S4. *R*-huezole demixes into spherical puncta. *R*-huezole (25 μM) was incubated with Nile Red (100 nM) in PBS buffer then imaged at indicated time intervals using 561 nm bandwidth filter by confocal microscopy. Scale bars, 35 μm.

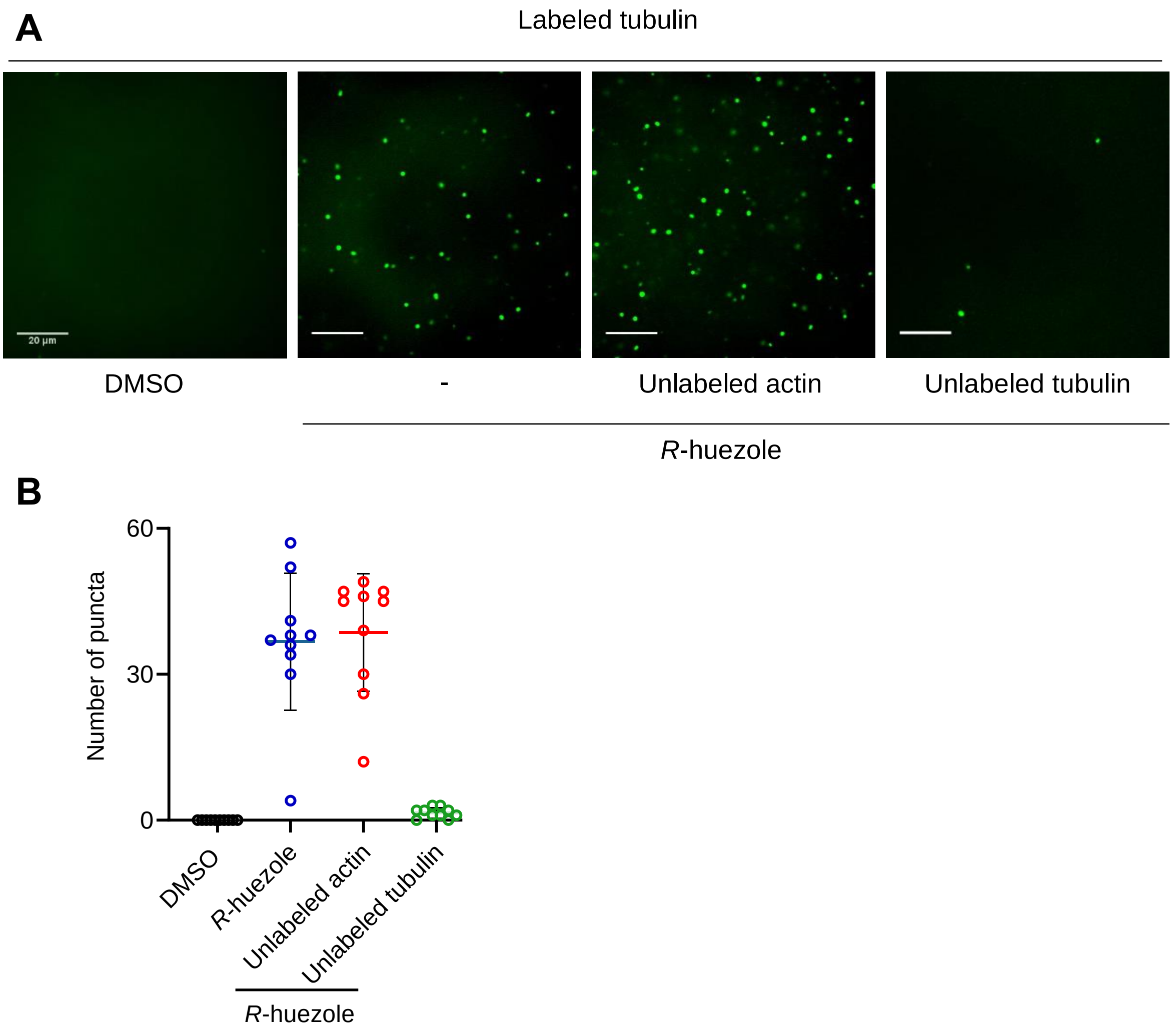

Figure S5. *In vitro* competition experiment. (A) Microscopic observation of labeled tubulin bound to *R*-huezole assemblies. *R*-huezole (50 µM) was incubated with excess concentration (4x) of unlabeled tubulin and actin (800 nM) for 30 min, followed by adding labeled tubulin (200 nM) for 30 min before imaging by confocal microscopy. (B) Quantification of the observed puncta in 10 microscopic fields. Data represents the mean ± S.D for two independent experiments.

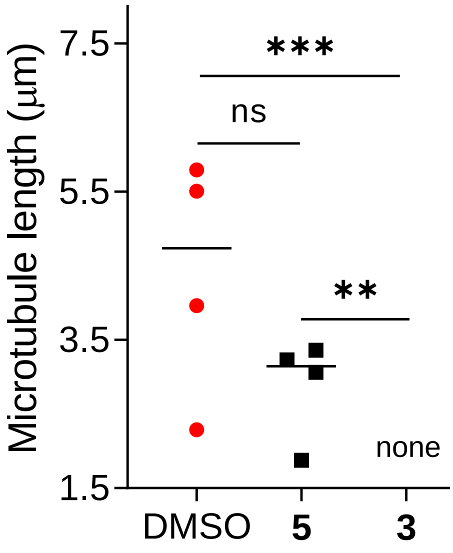

Figure S6. Quantification of Figure 4B. The length of the microtubules was quantified by Image J. The images were examined for fibres having length over 1.5 μm. Data represents average values mean ± S.D (n=2). No fibres were observed in the **3**-treated sample. The *P* values (***p*=0.0075 and ****p*=0.0004) were determined using one-way ANOVA followed by Tukey's multiple comparisons test with a 95% confidence interval.

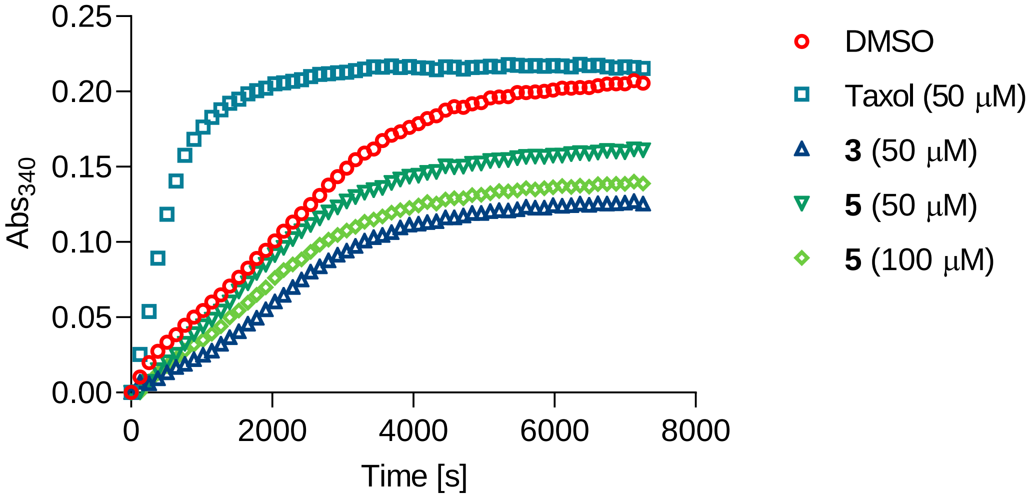

Figure S7. Inhibition of tubulin polymerization by **3** and **5**. Tubulin polymerization was monitored in the presence of **3** (50 μM), **5** (50 μM, 100 μM) and Taxol (50 μM) at 37°C using a kinetic measurement setting as described by the manufacturer. Data represents two independent experiments with similar results.

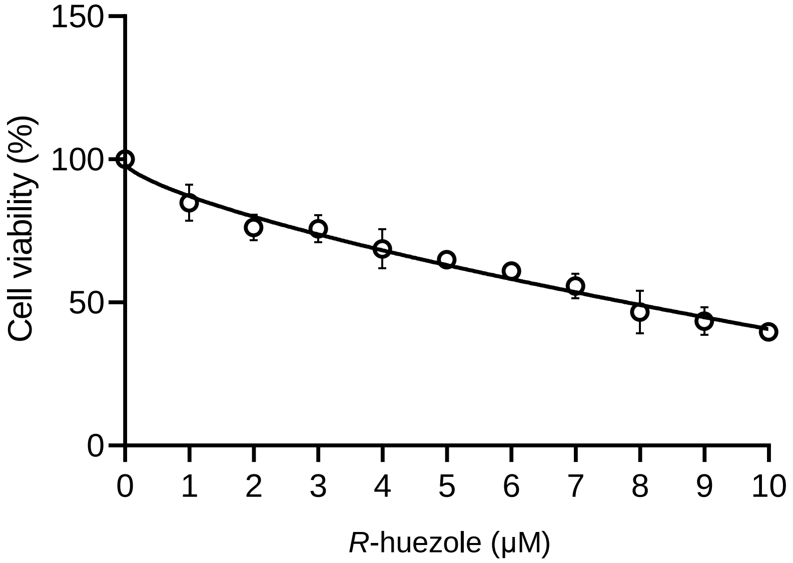

Figure S8. Growth inhibition of HeLa cells by *R*-huezole. HeLa cells were treated with DMSO (0.5%(v/v)) or *R*-huezole for 48 h, followed by cell viability analysis by WST-8 assays. The IC_{50} value was calculated to be 4.4 μM. Data represents mean ± S.D for three independent experiments.

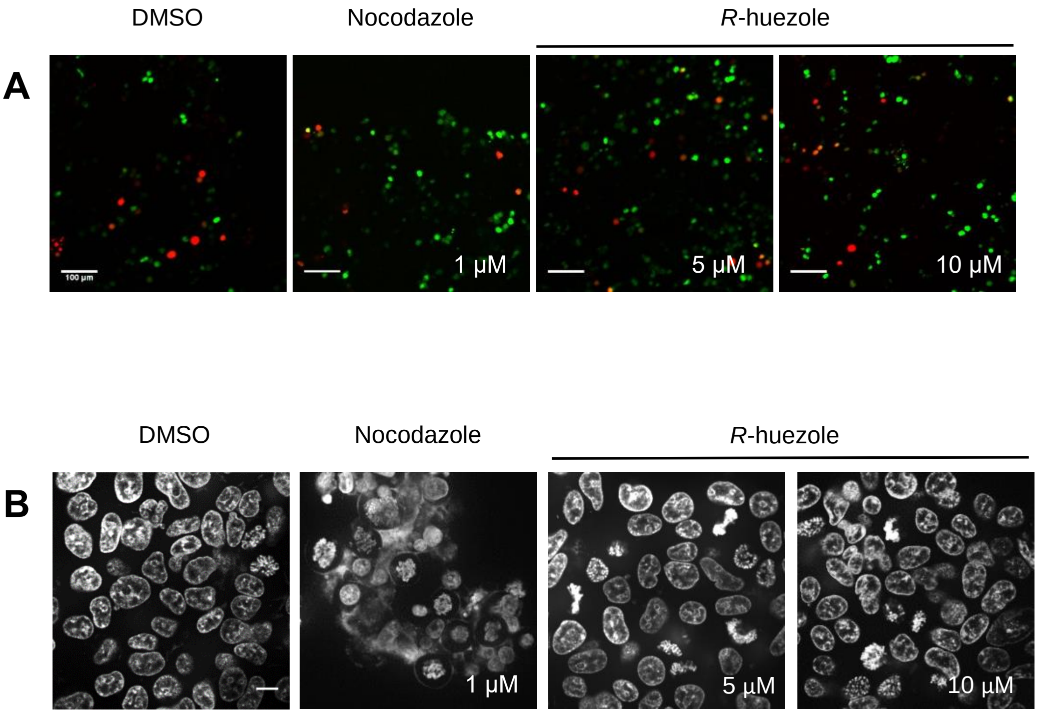

Figure S9. *R*-huezole leads to cell cycle arrest in HEK293 cells. (A) HEK293 cells expressing Fucci fluorescent probes, mCherry-hCdt1 (red) and AmCyan-hGeminin (green), were treated with DMSO, Nocodazole, or *R*-huezole (5 μM and 10 μM) for 48 h prior to live imaging by confocal microscopy. An increase in geminin/Cdt1 ratio indicates an increase in the cell population in the G2/M phase of the cell cycle. Nocodazole (1 μM) was used as a positive control. Data from three independent experiments from at least 10 microscopic fields representing at least 800 cells per condition. Scale bars, 100 μm. (B) Chromatin condensation observed in HEK293 cells treated with *R*-huezole. HEK293 cells were treated with DMSO, Nocodazole, or *R*-huezole (5 μM and 10 μM), followed by 48 h incubation. Nuclei were stained with Hoechst 33342 prior to live imaging by confocal microscopy. Scale bars, 10 μm. Data from three independent experiments from 9 microscopic fields representing at least 600 cells per condition.

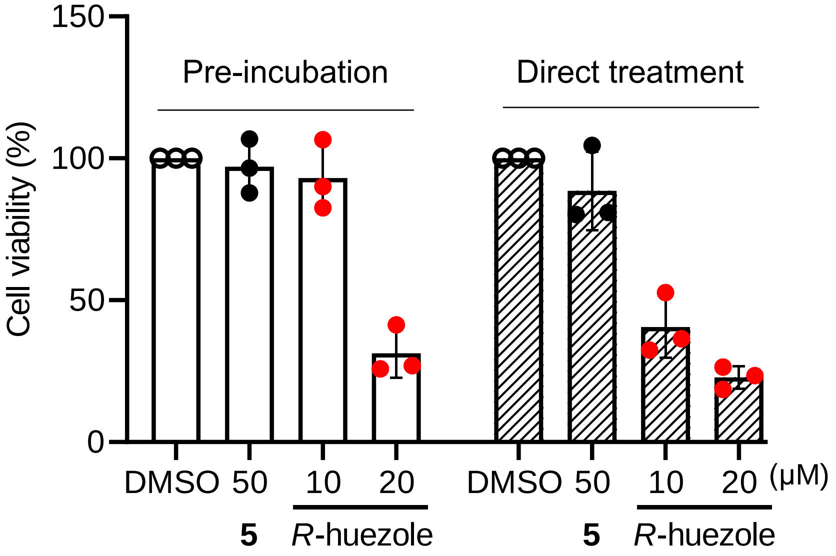

Figure S10. Effects of *R*-huezole (**3**) on HeLa cell growth. HeLa cells (5x10^3) were seeded onto a 96-well plate. DMSO (0.5%(v/v)), **5**, or *R*-huezole (**3**) were pre-incubated in media for 5 min before exposure to HeLa cells or directly exposed to the cells. Cell viability was analyzed after 48 h by WST-8 assays. Data represents mean ± S.D for three independent experiments.

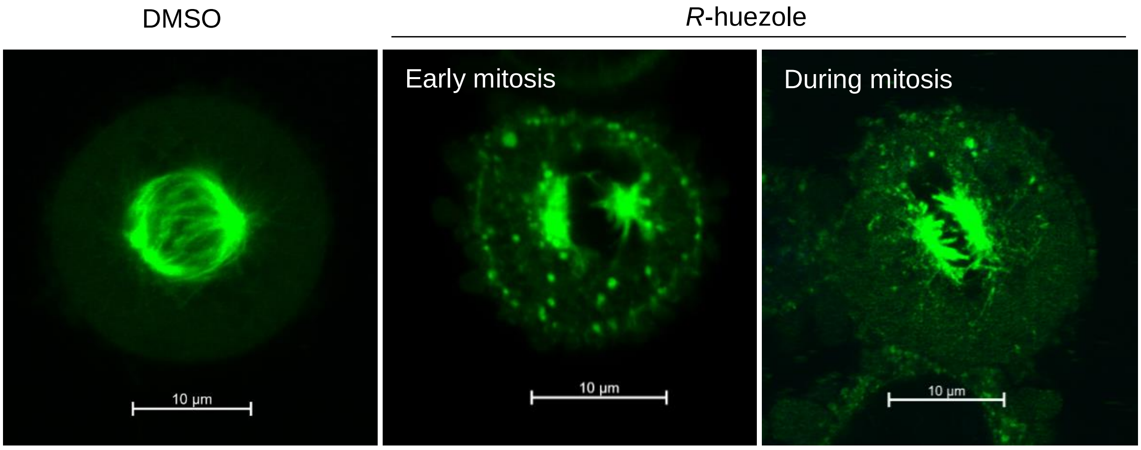

Figure S11. Effect of *R*-huezole on mitotic cells. HeLa cells were transduced with tubulin-GFP for 24 h followed by treatment with DMSO (1%(v/v)) or *R*-huezole (50 μM) for 1 h prior to imaging by Airyscan LSM-880. Scale bars, 10 µm

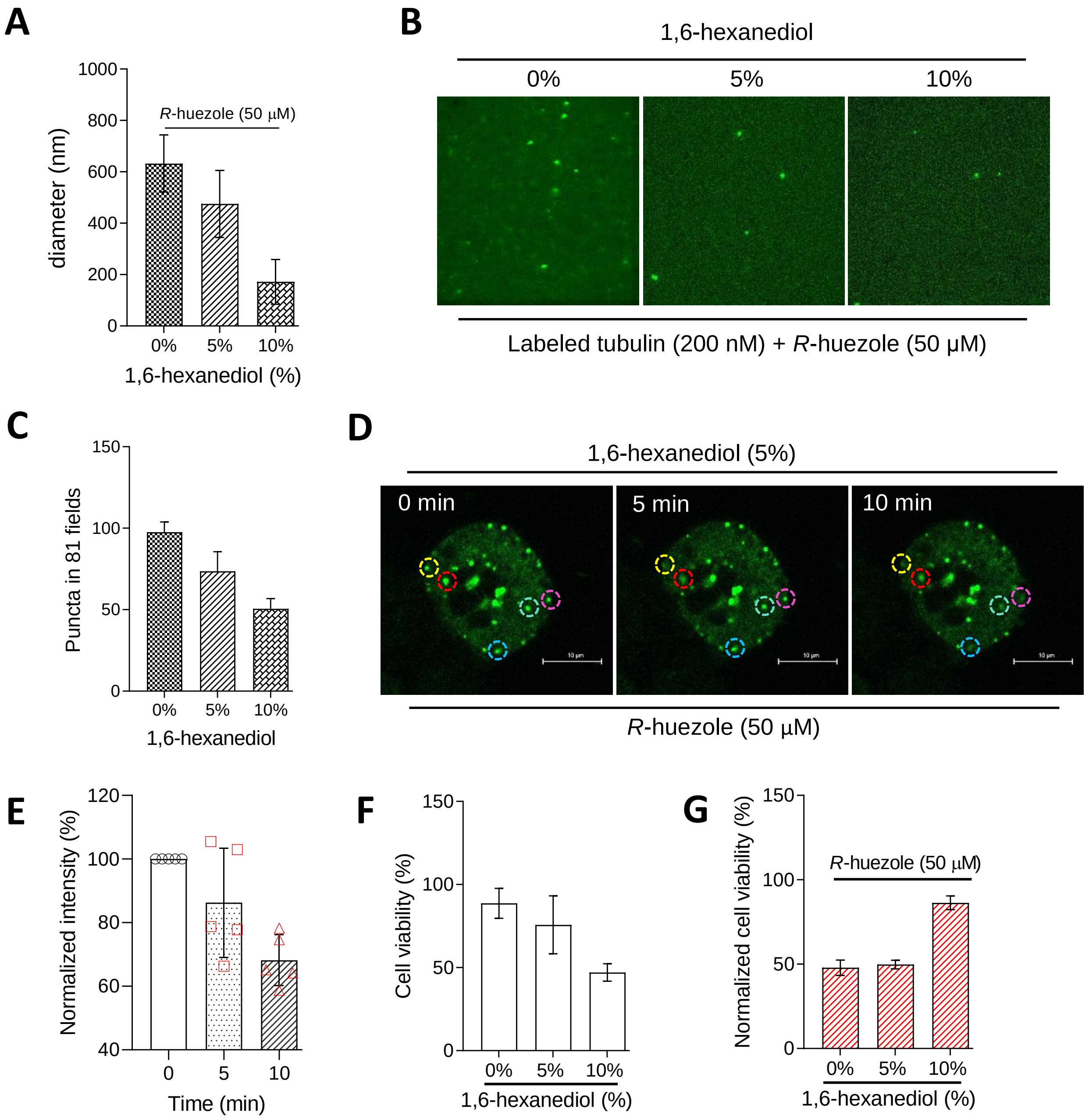

Figure S12. 1,6-hexanediol disrupts *R*-huezole puncta. Effect of 1,6-hexanediol on *R*-huezole and tubulin sequestration (A) Average hydrodynamic diameters of *R*-huezole at increasing concentrations of 1,6-hexanediol measured by DLS. The data indicates that particle formation propensity of *R*-huezole was disrupted by 1,6-hexanediol. Data represents average values mean ± SD (n = 3). (B) Labeled tubulin (200 nM) and *R*-huezole (50 μM) was incubated with increasing concentrations of 1,6-hexanediol (0%, 5%, 10%) followed by imaging by Airyscan LSM-880. Data represents average values mean ± SD (n = 2). (C) Quantification of (B). (D) Effect of 1,6-hexanediol on *R*-huezole treated HeLa cells. HeLa cells were transduced with Tubulin-GFP for 30 h followed by treatment with *R*-huezole (50 μM) for 1 h prior to addition of 1,6-hexanediol (5%). HeLa cells were monitored for dissolving of *R*-huezole condensates after treatment with 1,6-hexanediol (5%) at 5 min intervals by Airyscan LSM-880. Scale bars, 10 μm (E) Quantification of fluorescent intensities of the highlighted puncta from (D). (F) Effect of 1,6-hexanediol on HeLa cells. HeLa cells ($5x10^3$) were treated with varied concentrations of 1,6-hexanediol (0%, 5%, 10%) for 1 h prior to cell viability measurement. Data represents average values mean ± SD (n = 3). (G) Effect of *R*-huezole on 1,6-hexanediol-treated HeLa cells. HeLa cells ($5x10^3$) were treated with *R*-huezole (50 μM) for 1 h followed by addition of varied concentrations of 1,6-hexanediol (0%, 5%, 10%) for 1 h prior to cell viability measurement. Data were normalized to the cytotoxicity of 1,6-hexanediol for respective values of (F). Data represents average values mean ± SD (n = 3).

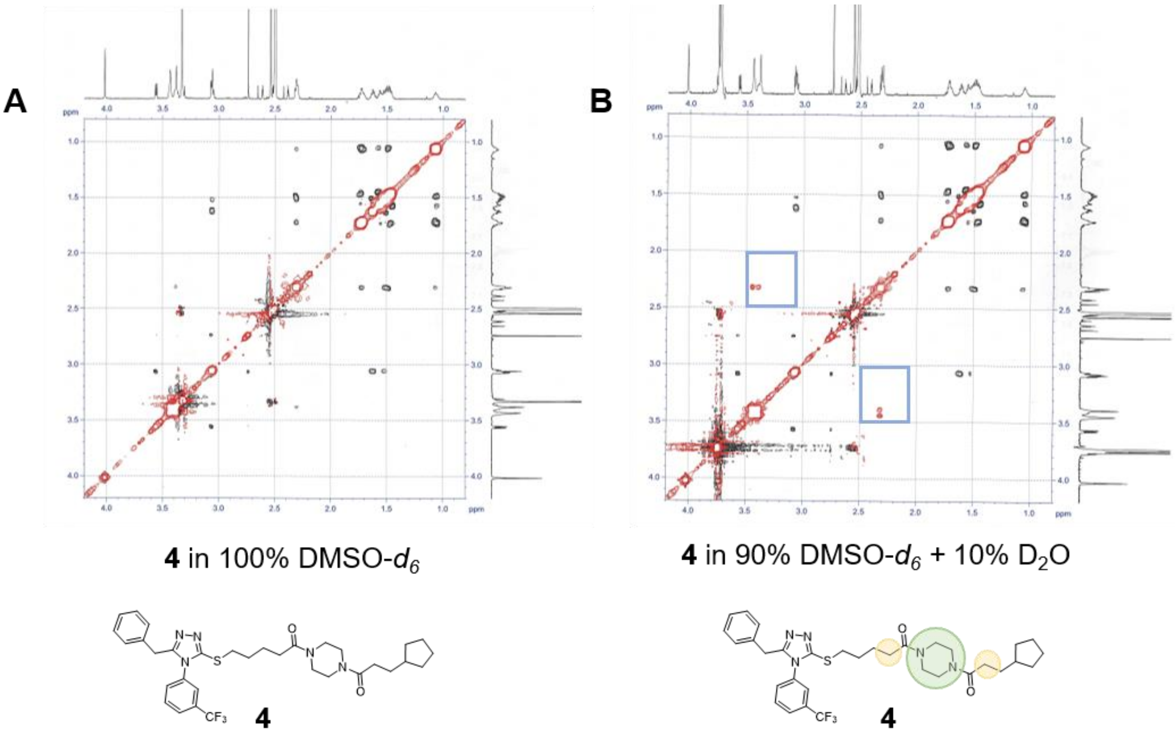

Figure S13. The modular structure of huezole. (A) ^{1}H-NOESY spectra of molecule **4** in 100% DMSO-d_6 or 90% DMSO-d_6 + 10% D_2O. Positive and negative NOE cross-peaks are indicated in black and red, respectively. Extra negative NOEs are indicated in blue boxes.

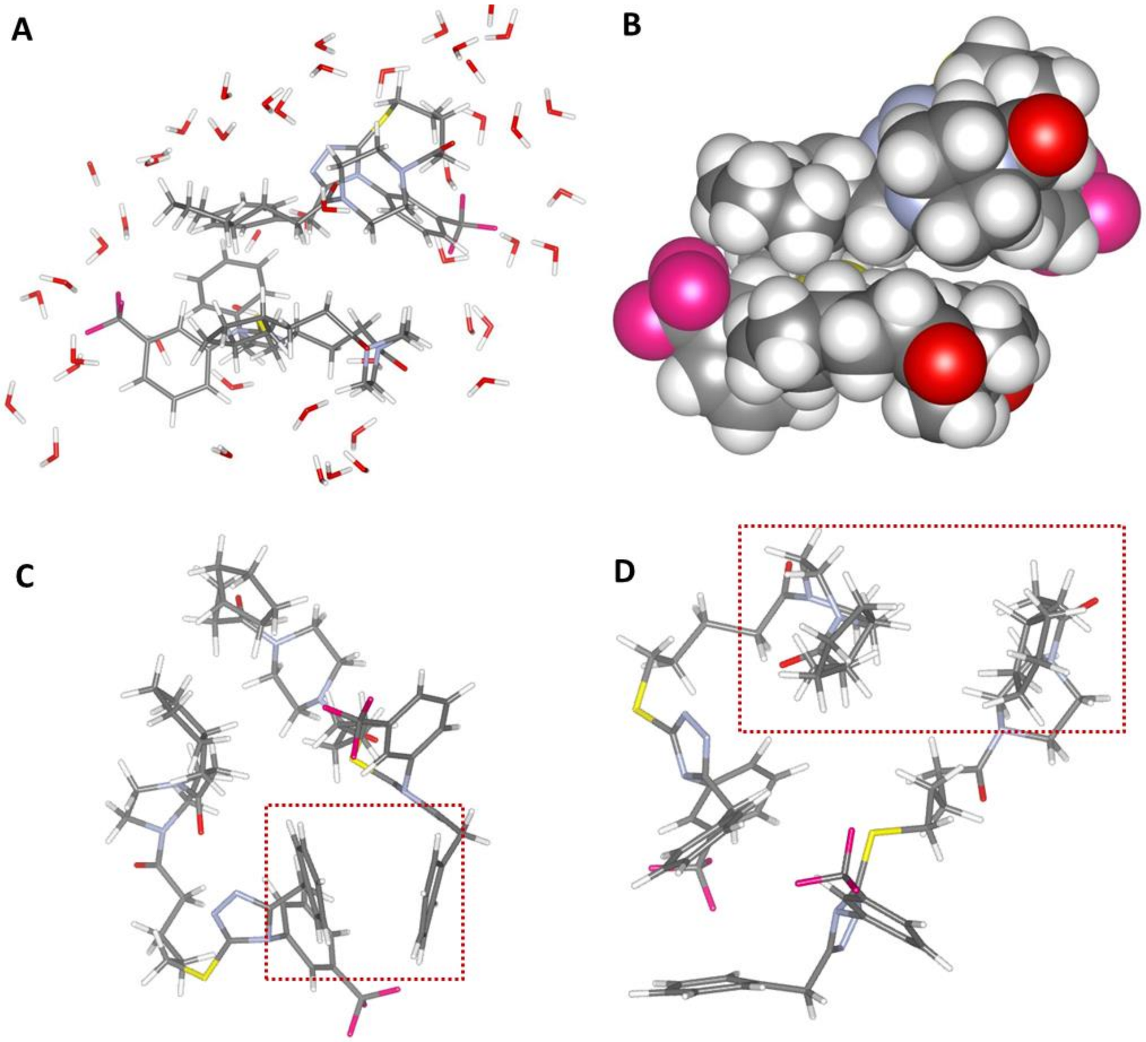

Figure S14. Molecular dynamics simulation of two huezole molecules (molecule **4**) in aqueous solvent. (A) Snapshot after 10 ns simulation. Only water molecules within 5 Å distance of the huezole molecules are shown. No clear coordination between the huezole molecules and solvent molecules can be seen. (B) As for A, but drawn with a space-filling model with water molecules removed from image. Both snapshots are viewed towards the interacting piperazine moieties. (C) and (D) As for B, but drawn as a stick representation and viewed from different angles to highlight hydrophobic interactions between phenyl moieties (C) and piperazine moieties (D). Grey = carbon, white = hydrogen, red = oxygen, blue = nitrogen, yellow = sulfur, pink = fluorine.

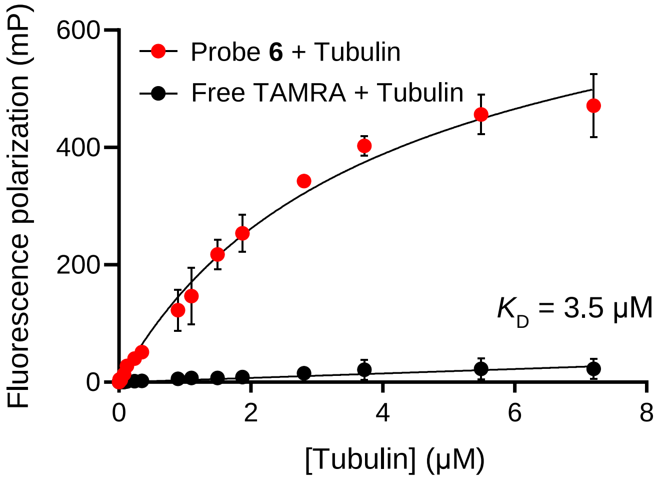

Figure S15. Fluorescence polarization (FP) measurements of probe **6** (100 nM) with increasing concentrations of tubulin. K_D was determined to be 3.5 μM by non-linear curve fitting with a one-binding-site model using GraphPad Prism. Free TAMRA probe (100 nM) displayed no detectable binding. Data represents mean ± S.D for three independent experiments.

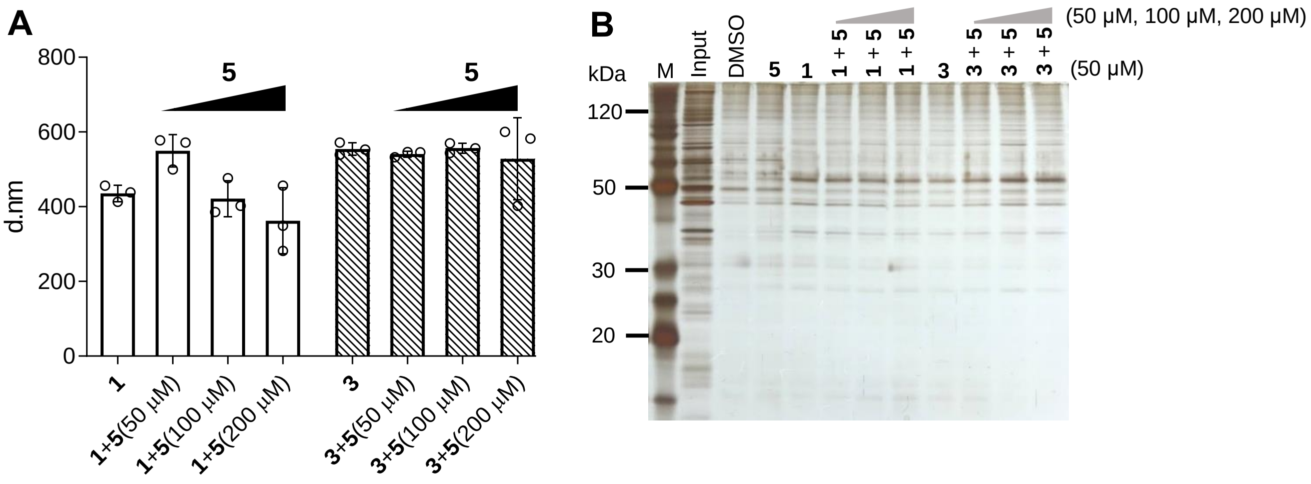

Figure S16. Effect of molecule **5** on particle formation and tubulin binding in presence of molecules **1** and **3**. (A) Average hydrodynamic diameters of **1** and **3** at increasing concentrations of **5** measured by DLS. The data indicates that particle formation propensity of **1** and **3** is not influenced by **5**. Data represents mean ± SD (n = 3) (B) Co-precipitation experiment of tubulin protein at same concentration conditions as Fig. S16(A). The co-precipitated tubulin bands displaying comparable band intensities affirming that molecule **5** neither promotes nor inhibits tubulin binding.

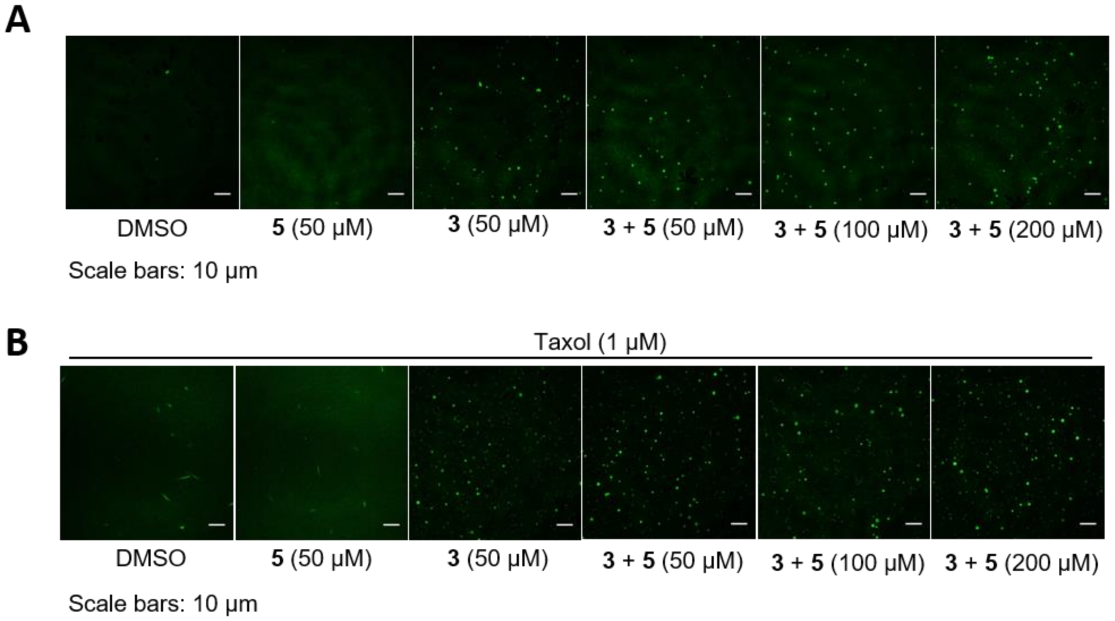

Figure S17. Effect of molecule **5** on *R*-huezole puncta and on tubulin polymerization *in vitro*. (A) Increasing concentration of **5** (50 µM, 100 µM, 200 µM) was incubated with labeled tubulin (200 nM) and *R*-huezole (50 µM) in PBS at RT for 30 min followed by imaging for the puncta formation by confocal microscopy. (B) In a tubulin polymerization condition (80 mM PIPES, pH 6.9, 2 mM $MgCl_2$, 1 mM GTP, and 0.5 mM EGTA), the effect of **5** was monitored on microtubule formation. Increasing concentration of **5** (50 µM, 100 µM, 200 µM) was incubated with labeled tubulin (200 nM) and *R*-huezole (50 µM) in the presence of taxol (1 µM). The samples were incubated at 37°C for 30 min prior to imaging by confocal microscopy.

Chemical Synthesis

All the compounds were synthesized according to the following synthetic scheme.

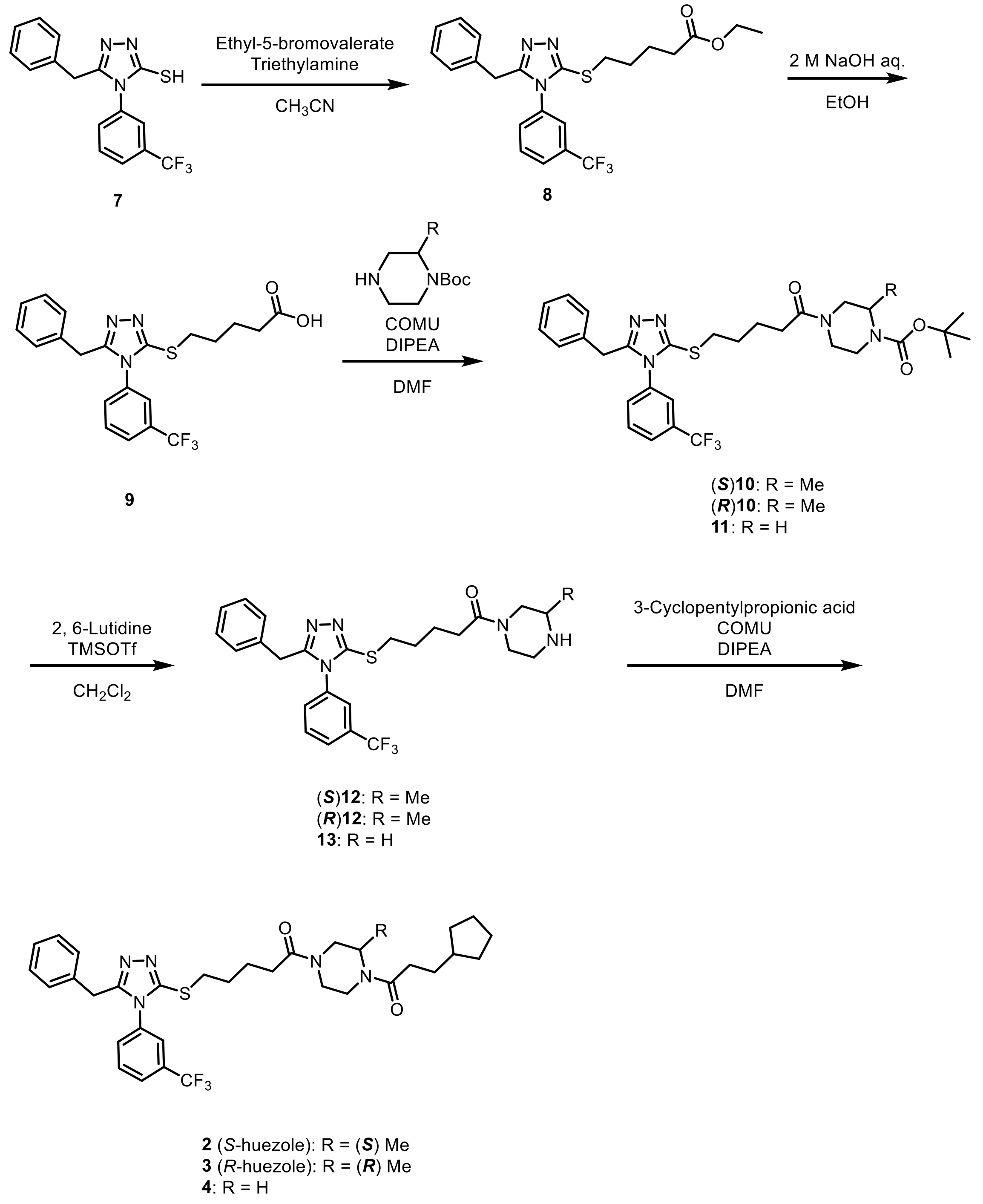

Scheme 1

Synthesis of Molecule 8

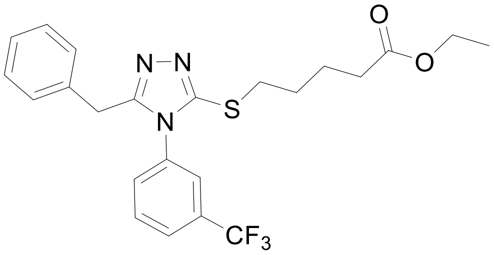

Compound **7** was synthesized according to the reported method[1] and used as a starting material. To a solution of compound **7** (1.12 g, 3.33 mmol) and triethylamine (2 mL, 14.3 mmol) in acetonitrile (20 mL), ethyl-5-bromovalerate (800 μL, 5.05 mmol) was added, and the mixture was stirred at room temperature for 4 h. The mixture was evaporated and extracted with EtOAc and applied to a silica gel column (hexane/EtOAc) to yield compound **8** as white solid (1.53 g, 99%). ^{1}H NMR (600 MHz, DMSO-d$_6$) δ: 1.16(t, 3H, J = 7.2 Hz), 1.51-1.57 (m, 2H), 1.62 (quin, 2H, J = 7.2 Hz), 2.27(t, 2H, J = 7.2 Hz), 3.05(t, 2H, J = 7.2 Hz), 4.02 (s, 2H), 4.03(t, 2H, J = 7.2 Hz), 6.88 (dd, 2H, J = 7.2, 1.8 Hz), 7.13-7.17 (m, 3H), 7.60 (m, 1H), 7.61 (m, 1H), 7.72 (tt, 1H, J = 7.8, 0.6 Hz), 7.88 (dquin, 1H, J =7.8 , 0.6 Hz), ^{13}C NMR (150 MHz, DMSO-d$_6$) δ: 13.99, 23.11, 28.19, 30.90, 31.96, 32.73, 59.62, 123.22(q, J = 271.5 Hz), 124.46(q, J = 4.5 Hz), 126.51, 126.54(q, J = 4.5 Hz), 128.20, 128.29, 130.18(q, J = 31.5 Hz), 130.91, 131.70, 133.70, 135.35, 150.06, 154.37, 172.51, ESI-HRMS (*m/z*): calc'd for $[C_{23}H_{25}F_3N_3O_2S]^+$ $[M+H]^+$: 464.1624, found 464.1612.

Synthesis of Molecule 9

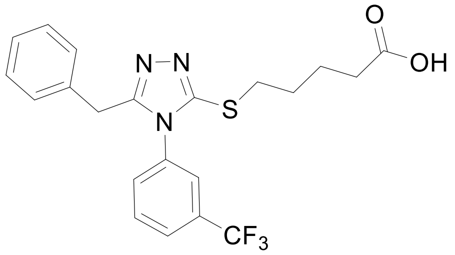

To a solution of compound **8** (1.52 g, 3.28 mmol) in ethanol (15 mL), 2 M NaOH aq (4 mL) was added, and the mixture was stirred at room temperature overnight. The reaction was quenched with addition of 1 M HCl aq and extracted with DCM to yield compound **9** as white solid (1.25 g, 88%). ^{1}H NMR (600 MHz, DMSO-d$_6$) δ: 1.52 (quin, 2H, J = 7.2 Hz), 1.63 (quin, 2H, J = 7.2 Hz), 2.20(t, 2H, J = 7.2 Hz), 3.06(t, 2H, J = 7.2 Hz), 4.03 (s, 2H), 6.88 (dd, 2H, J = 7.2, 1.8 Hz), 7.14-7.17 (m, 3H), 7.60-7.62 (m, 2H), 7.72 (t, 1H, J = 7.8 Hz), 7.87-7.89 (m, 1H), 12.04 (s, 1H), ^{13}C NMR (150 MHz, DMSO-d$_6$) δ: 23.22, 28.35, 30.91, 32.01, 32.90, 123.23(q, J = 271.5 Hz), 124.47(q, J = 4.5 Hz), 126.52, 126.54(q, J = 4.5 Hz), 128.21, 128.31, 130.22(q, J = 31.5 Hz), 130.92, 131.70, 133.71, 135.36, 150.12, 154.37, 174.12, ESI-HRMS (*m/z*): calc'd for $[C_{21}H_{19}F_3N_3O_2S]^-$ $[M-H]^-$: 434.1156, found 434.1154

Synthesis of Molecule (*S*)10

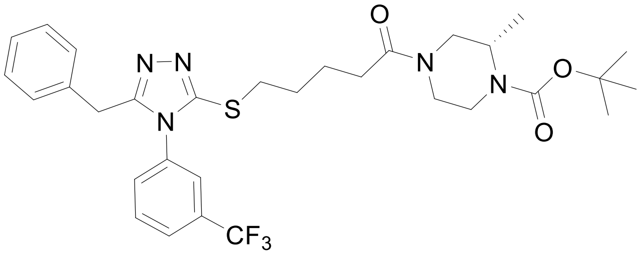

To a solution of compound **9** (200 mg, 0.46 mmol), tert-butyl (*S*)-2-methylpiperazine-1-carboxylate (92 mg, 0.46 mmol), and DIPEA (156 µL, 0.92 mmol) in DMF (2 mL), COMU (197 mg, 0.46 mmol) was added, the mixture was stirred at 0°C for 2 h and then at room temperature overnight. The reaction mixture was diluted with sat. $NaHCO_3$ aq., neutralized with diluted HCl and finally extracted with ethyl acetate. Then ethyl acetate layer was concentrated under reduced pressure to obtain a semi-solid residue. We used compound (***S***)**10** for the next step reaction without further purification.

Synthesis of Molecule (*R*)10

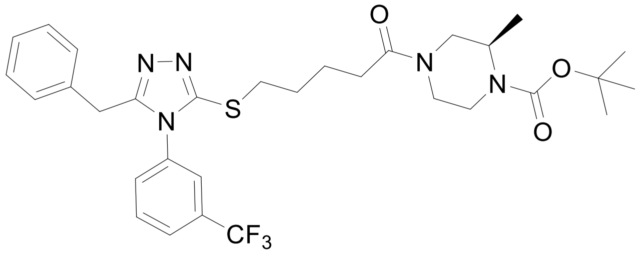

To a solution of compound **9** (870 mg, 2.00 mmol), tert-butyl (*R*)-2-methylpiperazine-1-carboxylate (400 mg, 2 mmol) and DIPEA (679 µL, 3.99 mmol) in DMF (10 mL), COMU (855 mg, 2.00 mmol) was added, and the mixture was stirred at 0°C for 2 h and then at room temperature overnight. The reaction mixture was diluted with sat. $NaHCO_3$ aq., neutralized with diluted HCl and finally extracted with ethyl acetate. Then ethyl acetate layer was concentrated under reduced pressure to obtain a semi-solid residue. We used compound (***R***)**10** for the next step reaction without further purification.

Synthesis of Molecule 11

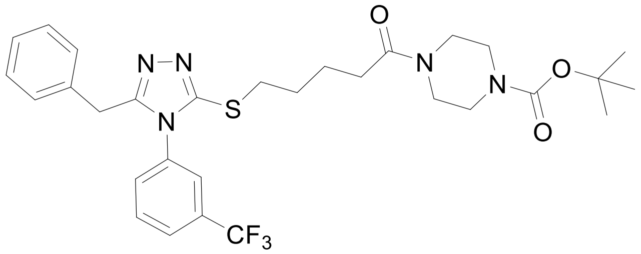

To a solution of compound **9** (1510 mg, 3.47 mmol), tert-butyl piperazine-1-carboxylate (1420 mg, 7.63 mmol) and DIPEA (1180 µL, 6.94 mmol) in DMF (10 mL), COMU (1630 mg, 3.82 mmol) was added, the mixture was stirred at 0°C for 2 h and then at room temperature overnight. The reaction mixture was diluted with sat. $NaHCO_3$ aq., neutralized with diluted HCl and finally extracted with ethyl acetate. Then ethyl acetate layer was concentrated under reduced pressure to obtain a semi-solid residue. We used compound **11** for the next step reaction without further purification.

Synthesis of Molecule (*S*)12

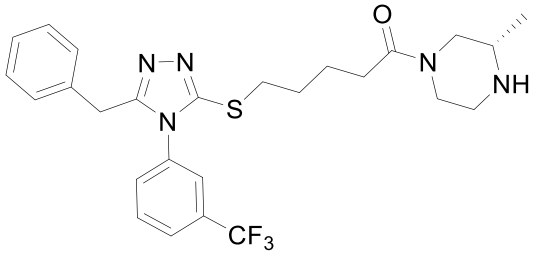

To a solution of compound (***S***)**10** (0.46 mmol), 2,6-lutidine (105 μL, 0.90 mmol) in dry DCM (2 mL), TMSOTf (98 μL, 0.54 mmol) was added at 0°C under argon gas atmosphere for 1 h. The mixture was then stirred at room temperature for 2 h. The reaction was quenched with sat $NaHCO_3$ aq and extracted with DCM. Then the DCM layer was washed with brine. Finally, the solution was concentrated under reduced pressure, characterized by LC/MS and used for next step reaction without further purification.

Synthesis of Molecule (*R*)12

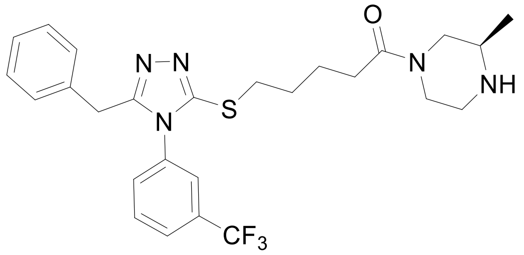

To a solution of compound (***R***)**10** (2 mmol), 2,6-lutidine (1526 μL, 13.11 mmol) in dry DCM (10 mL), TMSOTf (1169 μL, 6.47 mmol) was added at 0°C under argon gas atmosphere for 1 h. The mixture was then stirred at room temperature for 2 h. The reaction was quenched with saturated $NaHCO_3$ solution and extracted with DCM. Then the DCM layer was washed with brine. Finally, the solution was concentrated under reduced pressure, characterized by LC/MS and used for next step reaction without further purification.

Synthesis of Molecule 13

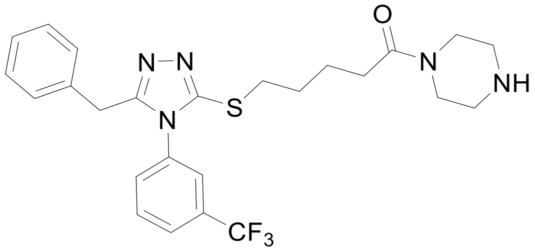

To a solution of compound **11** (3.47 mmol), 2,6-lutidine (2485 μL, 21.33 mmol) in dry DCM (15 mL), TMSOTf (1903 μL, 10.53 mmol) was added at 0°C under argon gas atmosphere for 1 h. The mixture was then stirred at room temperature for 2 h. The reaction was quenched with saturated $NaHCO_3$ solution and extracted with DCM. Then the DCM layer was washed with brine. Finally, the solution was concentrated under reduced pressure, characterized by LC/MS and used for next step reaction without further purification.

Synthesis of Molecule 2 (*S*-huezole)

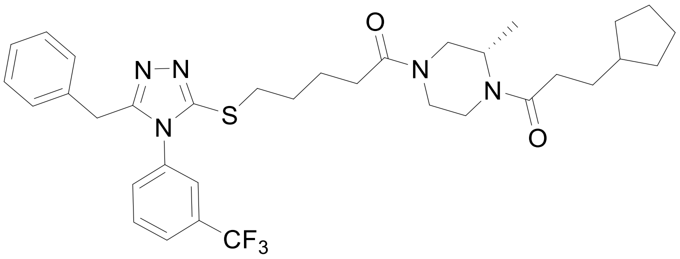

To a solution of compound (***S***)**12** (0.46 mmol), DIPEA (156 μL, 0.92 mmol) and 3-cyclopentylpropionic acid (67 μL, 0.48 mmol) in dry DMF (2 mL), COMU (197 mg, 0.46 mmol) was added at 0°C for 1 h. The mixture was then stirred at room temperature overnight. The reaction was quenched with sat $NaHCO_3$ aq and extracted with EtOAc. Then EtOAc layer was concentrated under reduced pressure and applied to a silica gel column (MeOH/$CHCl_3$ = 1/10 (v/v)) to yield compound **2** as yellow oil (121.7 mg, 41% in 3 steps). ^{1}H NMR (600 MHz, DMSO-*d*$_6$) δ: 0.90-1.11 (m, 4H), 1.39 (s, 2H), 1.45-1.71 (m, 10H), 2.23-2.38 (m, 3H), 2.58-3.24 (m, 4.5H), 3.61-3.68(m, 1.5H), 3.76-3.78(m, 0.5H), 4.01 (s, 2H), 4.07-4.16 (m, 2H), 4.52 (bs, 0.5H), 6.86 (dd, 2H, *J* = 7.2, 1.8 Hz), 7.13-7.14 (m, 3H), 7.55 (s , 1H), 7.67 (d, 1H, *J* = 7.8 Hz), 7.72 (t, 1H, *J* = 7.8 Hz), 7.87 (d, 1H, *J* =7.8 Hz), ^{13}C NMR (151 MHz, DMSO-*d*$_6$) δ:14.47, 14.57, 14.87, 15.01, 15.47, 15.82, 23.37, 23.40, 23.41, 24.55, 27.90, 28.39, 30.84, 31.00, 31.12, 31.23, 31.32, 31.36, 31.85, 31.94, 31.99, 32.06, 35.22, 35.81, 39.79, 40.07, 40.80, 41.22, 44.03, 44.08, 44.44, 44.54, 44.75, 45.04, 46.60, 47.91, 48.36, 48.91, 78.96, 78.99, 79.00, 120.46, 122.26, 124.07, 123.39(q, *J* = 4.5 Hz), 125.88, 126.52, 126.56, 128.20, 128.24, 130.18(q, *J* = 33.0 Hz), 130.93, 131.64, 133.61, 135.26, 150.20, 153.65, 153.68, 154.36, 170.89, 170.96, ESI-HRMS (*m/z*): calc'd for $[C_{34}H_{42}F_3N_5NaO_2S]^+$ $[M+Na]^+$: 664.2904, found 664.2904.

Synthesis of Molecule 3 (*R*-huezole)

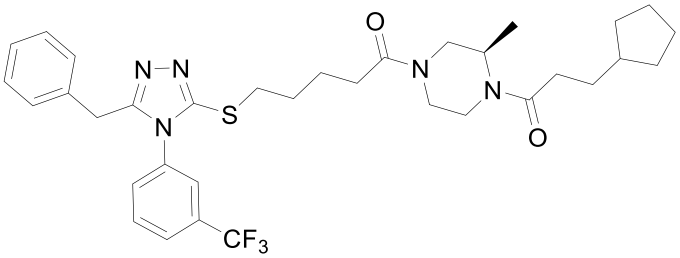

To a solution of compound (***R***)**12** (2 mmol), DIPEA (266 μL, 1.56 mmol) and 3-cyclopentylpropionic acid (220 μL, 1.56 mmol) in dry DMF (5 mL), COMU (670 mg, 1.56 mmol) was added at 0°C for 1 h. The mixture was then stirred at room temperature overnight. The reaction was quenched with sat $NaHCO_3$ aq and extracted with EtOAc. Then EtOAc layer was concentrated under reduced pressure and applied to a silica gel column (MeOH/$CHCl_3$ = 1/10 (v/v)) to yield compound **3** as yellow oil (320 mg, 32% in 3 steps). ^{1}H NMR (600 MHz, DMSO-*d*$_6$) δ: 0.91-1.08 (m, 5H), 1.18 (t, 0.4H, *J* = 7.2 Hz), 1.26 (dt, 0.4H, *J* = 8.4, 6.6 Hz), 1.46-1.72 (m, 13H), 2.23-2.42 (m, 4H), 2.67-3.25 (m, 4.5H), 3.64-3.83(m, 1.5H), 4.01-4.27 (m, 2H), 4.02 (s, 2H), 4.53 (bs, 0.5H), 6.87 (dd, 2H, *J* = 7.2, 1.8 Hz), 7.13-7.17 (m, 3H), 7.59 (s , 1H), 7.61 (d, 1H, *J* = 7.8 Hz), 7.72 (t, 1H, *J* = 7.8 Hz), 7.88 (d, 1H, *J* =7.8 Hz), ^{13}C NMR (151 MHz, DMSO-*d*$_6$) δ:12.41, 13.98, 14.50, 14.91, 15.49, 15.85, 16.62, 17.98, 20.66, 23.40, 23.43, 24.57, 28.44, 30.83,30.88, 31.01, 31.13, 31.24, 31.33, 31.85, 31.96, 32.01, 32.09, 35.24, 35.82, 40.07, 41.22, 44.00, 44.05, 44.54, 44.76, 45.04, 47.88, 48.36, 48.92,

53.49, 54.82, 59.66, 120.49, 122.30, 124.10, 124.45(q, *J* = 4.5 Hz), 125.91, 126.50, 126.54(q, *J* = 3.6 Hz), 128.19, 128.28, 130.18(q, *J* = 33.0 Hz), 130.91, 131.68, 133.69, 135.34, 150.13, 154.32, 170.25, 170.74, 170.82, 170.85, ESI-HRMS (*m/z*): calc'd for $[C_{34}H_{43}F_3N_5O_2S]^+$ $[M+H]^+$: 642.3084, found 642.3079.

Synthesis of Molecule 4

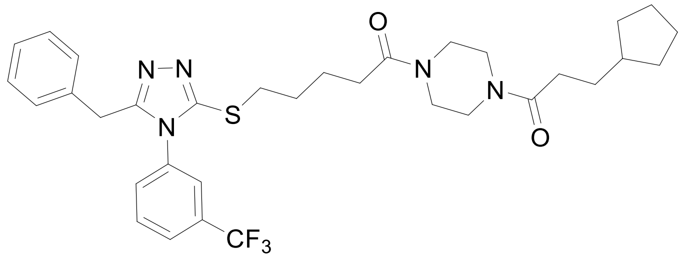

To a solution of compound **13** (3.47 mmol), DIPEA (332 μL, 1.95 mmol) and 3-cyclopentylpropionic acid (274 μL, 1.95 mmol) in dry DMF (5 mL), COMU (835 mg, 1.95 mmol) was added at 0°C for 1 h. The mixture was then stirred at room temperature overnight. The reaction was quenched with sat $NaHCO_3$ aq and extracted with EtOAc. Then EtOAc layer was concentrated under reduced pressure and applied to a silica gel column (MeOH/$CHCl_3$ = 1/10 (v/v)) to yield compound **4** as yellow oil (461.5 mg, 46% in 3 steps). ^{1}H NMR (600 MHz, DMSO-d_6) δ: 1.03-1.12 (m, 2H), 1.46-1.58 (m, 7H), 1.63 (quin, 2H, *J* = 7.8 Hz), 1.69-1.78 (m, 3H), 2.31 (t, 4H, *J* = 7.2 Hz), 2.74 (s, 2H), 3.06(d, 2H, *J* = 6.6 Hz), 3.38 (bs, 2H), 3.44 (bs, 2H), 4.02 (s, 2H), 6.87 (dd, 2H, *J* = 7.2, 1.8 Hz), 7.13-7.16 (m, 3H), 7.59 (s, 1H), 7.61 (d, 1H, *J* = 7.8 Hz), 7.72 (t, 1H, *J* = 7.8 Hz), 7.88 (d, 1H, *J* =7.8 Hz), ^{13}C NMR (151 MHz, DMSO-d_6) δ: 23.31, 24.57, 28.40, 28.44, 30.87, 30.90, 31.41, 31.45, 31.48, 31.56, 31.97, 32.07, 37.85, 39.93, 40.62, 40.66, 40.99, 41.07, 44.47, 44.55, 44.83, 44.88, 120.49, 122.30, 124.10, 124.44(q, *J* = 4.5 Hz), 125.91, 126.50, 126.53, 126.55(d, *J* = 4.5 Hz), 128.19, 128.27, 130.17(q, *J* = 31.5 Hz), 130.90, 131.68, 133.68, 135.33, ESI-HRMS (*m/z*): calc'd for $[C_{33}H_{41}F_3N_5O_2S]^+$ $[M+H]^+$: 628.2928, found 628.2929.

Synthesis of Molecule 5

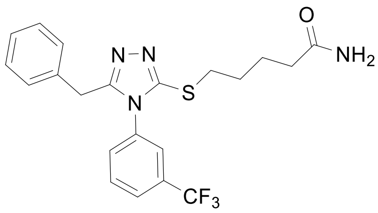

To a stirred mixture of DCC (28 mg, 0.136 mmoles), HOBT (18.376 mg, 0.136 mmoles) and DIPEA (42.5 μL, 0.25 mmoles) in DMF, compound **9** was added (50 mg, 0.114 mmoles) followed by addition of NH_4Cl (7.2mg, 0.136 mmoles) at room temperature. The reaction was stirred overnight. The reaction mixture was quenched with addition of ice and extracted in EtOAc. Then EtOAc layer was concentrated under reduced pressure and applied to a silica gel column (MeOH/DCM = 1/10 (v/v)) to yield compound **5** as semi-solid (25.5 mg, 50%). ^{1}H NMR (600MHz, DMSO-d_6) δ: 1.51 (quin, 2H, *J* = 7.2 Hz), 1.60 (quin, 2H, *J* = 7.2 Hz), 2.02(t, 2H, *J* = 7.2 Hz), 3.05(t, 2H, *J* = 7.2 Hz), 2.67-3.25 (m, 4.5H), 4.02 (s, 2H), 6.71 (bs, 1H), 6.87 (dd, 2H, *J* = 7.2, 1.8 Hz), 7.13-7.17 (m, 3H), 7.23 (bs, 1H), 7.60 (s , 1H), 7.61 (d, 1H, *J* = 7.8 Hz), 7.72 (t, 1H, *J* = 7.8 Hz), 7.88 (d, 1H, *J* =7.8 Hz), ^{13}C NMR (201MHz, DMSO-d_6) δ: 23.86, 28.51, 30.87, 31.97, 34.29, 48.49, 120.49, 122.30, 124.10, 124.40, 124.44(q, *J* = 4.5 Hz), 125.91, 126.50, 126.53(q, *J* = 4.5 Hz), 128.19, 128.28, 130.18(q, *J* = 33.0 Hz), 130.92,

131.67, 133.67, 135.33, 150.17, 154.32, 173.78, ESI-HRMS (*m/z*): calc'd for $[C_{21}H_{22}F_3N_4OS]^+$ $[M+H]^+$: 435.1461, found 435.1459.

Synthesis of Molecule 6 (probe 6)

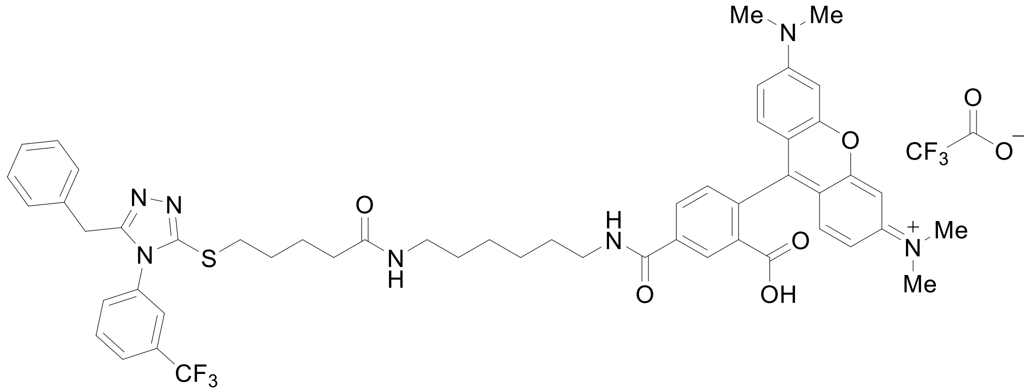

Compound **9** (0.66 mg, 1.525 mmol) and N-(9-(4-((6-aminohexyl)carbamoyl)-2-carboxyphenyl)-6-(dimethylamino)-3H-xanthen-3-ylidene)-N-methylmethanaminium (0.98 mg, 1.525 mmol) were dissolved in dry DMF (400 μL) at 0°C under argon gas atmosphere. To the reaction mixture, COMU (0.78mg, 1.83 mmol) and DIPEA (1.06 μl, 6.1 mmol) were added. The reaction mixture was stirred for 15 min at 0ºC. Completion of reaction was confirmed by LC/MS analysis. After completion of reaction, the solvent was removed by nitrogen gas flow. The compound was purified by HPLC using acetonitrile containing 0.1% TFA and water containing 0.1% TFA mixture as eluting solvent mixture. The titled compound was synthesized as described above and obtained as red solid, yield 68%. ESI-HRMS (*m/z*): calc'd for $[C_{52}H_{53}F_3N_7O_5S]^+$ $[M]^+$:946.3932, found 946.3930.

Experimental Procedures

Cell culture

HEK293 cells and HeLa cells were maintained in Dulbecco's modified Eagle Medium (DMEM, GIBCO) supplemented with 100 units/mL of penicillin, 100 μg/mL of streptomycin sulfate (Nacalai Tesque) and 10% fetal bovine serum (FBS, Biosera) at 37°C in a humidified 5% CO_2 incubator.

Materials and Methods

All the chemicals were purchased from Tokyo Chemical Industry, Sigma Aldrich and Wako Chemicals. For immunoblotting experiments, the proteins were resolved on a 12% SDS-PAGE acrylamide gel and transferred onto a nitrocellulose blotting membrane (GE Healthcare). The membranes were blocked for 1 h with 5% skim milk and incubated overnight with anti-alpha tubulin antibody (ab4074), anti-beta tubulin antibody (ab6046), anti-actin antibody AC-40 (ab11003), or anti-GAPDH antibody FL-335 (sc-25778). The membranes were washed three times with PBS-T, followed by 1 h incubation at room temperature with CST anti-rabbit IgG HRP-linked antibody (7074), or Sigma mouse IgG HRP-linked antibody (NA931V). The membranes were washed three times before adding ECL western blotting detection reagents (GE Healthcare). The bands were observed using an ImageQuant LAS 500 imaging system (GE Healthcare).

Screening

The screening method was adapted as described previously[2]. Confluent HeLa cells (10 cm dish) were collected and washed with ice cold 1x PBS, followed by freeze-thaw lysis. The cell lysates were then centrifuged (14000 rpm, 4°C, 30 min), and the supernatant fraction (50 µL) was used to incubate with library molecules (20 µM) at 4°C for 30 min using a Taitec rotator RT-50. The samples were centrifuged (14000 rpm, 4°C, 10 min) and washed thrice with PBS. The supernatant and pellet protein samples were mixed with 6x Sample Buffer Solution (Nacalai, product no. 09499-14), resolved on a 12% SDS-PAGE gel, and analysed by silver staining (Wako Silver Stain MS kit; 299-58901).

Dynamic Light Scattering (DLS)

The hydrodynamic diameters and correlation coefficients of molecules (**2**, **3**, **4** and **5**) were measured by Malvern Dynamic Light Scattering Spectrophotometer (Zetasizer Nano 1600) at 50 µM (final concentration) in PBS buffer at room temperature. All measurements were set to an auto-attenuation mode at 25°C and data was analysed from at least 10 acquisitions/measurement.

Nile Red Confocal Microscopy

Compounds at mentioned concentrations were added in PBS at room temperature followed by addition of Nile Red (100 nM). The time-lapse imaging was performed at every 20 seconds and 3 min intervals for Figure 3A and Figure S4 respectively. The size of self-assembly was visualized by CV1000-SP130 using 561 nm band-filter and bright field filter.

***In Vitro* Sequestration of Tubulin**

HiLyte Fluor™ 488-labelled tubulin (Cytoskeleton, Inc) (200 nM) was incubated with DMSO (1%(v/v)) or *R*-huezole (50 µM) in PBS for 30 min at room temperature prior to imaging. Confocal images were obtained using CV1000-SP130 (Cell Voyager CV1000, Yokogawa Electrical Corporation).

***In Vitro* Tubulin Polymerization Assay**

DMSO (1%(v/v)), **5** (50 µM), or *R*-huezole (50 µM) were incubated with HiLyte Fluor™ 488-labeled tubulin (200 nM) in tubulin buffer (BST01-001) containing 1 mM GTP (BST06-001) and 1 µM Taxol (Sigma-Aldrich; T7402) for 30 min at 37°C. The samples were then imaged using CV1000-SP130 (Cell Voyager CV1000, Yokogawa Electrical Corporation). For Fig. 4C, polymerization of labeled tubulin (900 nM) was induced overnight by Taxol (50 µM) in the presence or absence of 50 µM *R*-huezole (**3**) in PBS. While 200 nM of labeled tubulin was sufficient for the sequestration of labeled tubulin by *R*-huezole, a higher concentration of labeled tubulin (900 nM) was needed for inducing microtubule polymerization *in vitro*.

***In Vitro* Competition Assay**

Unlabeled tubulin (T240-A) or actin (AKL99-B) (800 nM) were incubated with 50 µM *R*-huezole in PBS for 30 min at 37°C, followed by 30 min incubation with labeled tubulin (200 nM) at 37°C prior to imaging by confocal microscope. The observed puncta were quantified using ImageJ (NIH).

WST-8 Assay

HeLa (5x10^3) cells were seeded onto a 96 well plate. After 24 h, cells were treated with DMSO (0.5%(v/v)) or compounds at various concentrations for 48 h. 10 µL of Cell Counting Kit-8 solution (DOJINDO; 343-07623) was added. Absorbance was measured after 1 h by Microplate Reader MTP-880 (CORONA) at 450 nm.

Proliferation Assay

This method was adapted as reported previously[3]. HeLa cells (25,000) were seeded onto 6-well plates in triplicates. After treatment with DMSO (0.05%(v/v)) or *R*-huezole (5 µM), cells were counted and re-seeded at 25,000 cells/well for 96 h, 192 h and 288 h intervals. Cells were counted by Countess™ automated cell counter (Invitrogen).

Fucci

HEK293 cells (1x10^4) were grown for 24 h prior to transfection with 100 ng of Fucci fluorescent probes mCherry-hCdt1 (30/120) (red) and AmCyan-hGeminin (green) as described[4]. 24 h after transfection, DMSO (0.5%(v/v)), Nocodazole (Sigma-Aldrich; M1404) (1 µM), or *R*-huezole (5 and 10 µM) were treated for 48 h. Images of cells in G1, S or G2/M phases were obtained by confocal microscope and quantified using ImageJ (NIH). Plasmids pMXs–mCherry–hCdh1 (30:120) and pCSII–AmCyan–hGeminin (1:110) were kindly provided by Prof. Atsushi Miyawaki (RIKEN BSI, Japan).

Live Cell Imaging of Tubulin-GFP

HeLa cells (5x10^3) were seeded onto a 96 well plate and transduced by tubulin-GFP baculovirus particles (CellLight™ Tubulin-GFP, BacMam 2.0; C10613) for 30 h. The cells were then treated with DMSO (0.5%(v/v)) or *R*-huezole (50 µM) for 3 h, followed by nuclei staining with Hoechst 33342 prior to imaging. Confocal images were obtained using a Zeiss Airyscan LSM-880. The image represents at least three independent experiments with similar results.

Simulation

The molecular dynamics simulation was performed using the LAMMPS package[5] and the DREIDING force field[6]. The simulation considered two copies of molecule **4** and 900 water molecules in a cubic box with periodic boundary conditions. Molecule geometries and box dimensions were relaxed before starting the simulation, and the box dimensions were 43.06 Å x 43.06 Å x 43.06 Å after relaxation. The simulation was ran for 10 ns with an NVT ensemble and a Nose-Hoover thermostat at a temperature of 300 K. Input files were prepared using an in-house script. Output files were visualised with the VESTA software[7], after processing with an in-house script.

Fluorescence Polarization

Probe **6** or free-TAMRA (Sigma-Aldrich; 760757) were added to PBS (100 nM final concentration) in a quartz cuvette. Tubulin was titrated into the quartz cuvette (0 - 7.2 µM) from stock solutions (1, 2, 5, or 10 µL). FP signal was optimized for fluctuations and then monitored for 3 mins per titration at room temperature using LS-55 fluorescence spectrophotometer (Perkin Elmer).

Absorbance-based Tubulin Polymerization Assay
DMSO (1% (v/v)), Taxol (50 μM), **3** (50 μM), or **5** (50 μM, 100 μM) were mixed with tubulin (2mg/mL) and the tubulin polymerization dynamics was measured using a kinetic setting by Tecan Infinite M200PRO as described by the manufacturer (Cytoskeleton, BK006P).

1,6-Hexanediol Experiment
For DLS analysis, huezole (**1**), *R*-huezole (**3**), or **5** were incubated in PBS containing increasing concentrations of 1,6-hexanediol (0%, 5%, 10%), followed by measurements for average hydrodynamic diameters using a Malvern Dynamic Light Scattering Spectrophotometer (Zetasizer Nano-S 1600). For confocal microscopy, labeled tubulin (200 nM) and *R*-huezole (50 μM) were incubated in increasing concentrations of 1,6-hexanediol (0%, 5%, 10%), followed by imaging using an Airyscan LSM-880 confocal microscope and quantification using ImageJ (NIH). For cell-based experiment, HeLa cells were transduced with tubulin-GFP for 30 h followed by treatment with *R*-huezole (50 μM) for 1 h. 5% of 1,6 hexanediol was added prior to imaging at 5 min intervals using an Airyscan LSM-880 confocal microscope and quantification by ImageJ (NIH).

1,6-Hexanediol Treated WST-8 Assay
HeLa cells ($5x10^3$) were seeded onto a 96 well plate. After 24 h, cells were treated with DMSO (1%(v/v)) or **3** (50 μM) for 1 h and then with increasing concentrations of 1,6-hexanediol (0%, 5%, 10%) for 1 h. 10 μL of Cell Counting Kit-8 solution (DOJINDO; 343-07623) was added. Absorbance was measured after 1 h by Microplate Reader MTP-880 (CORONA) at 450 nm.

Compounds NMR Data

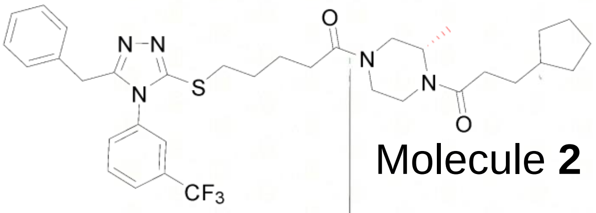

Molecule **2**

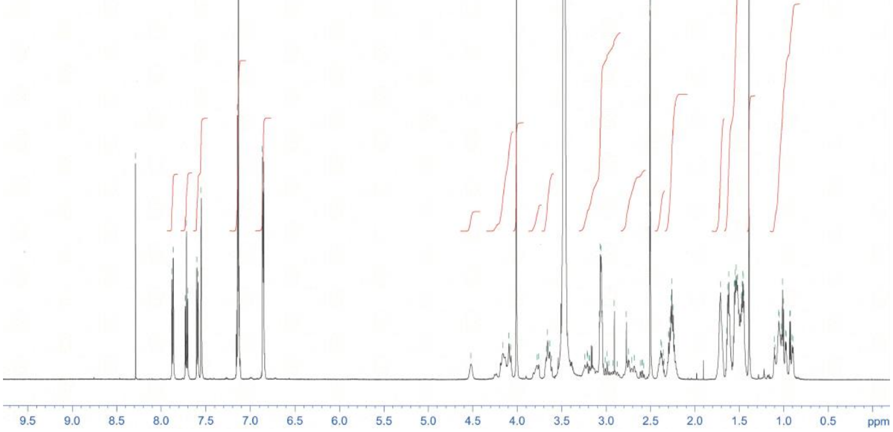

1H-NMR (600 MHz, 298.2 K), DMSO-*d6*

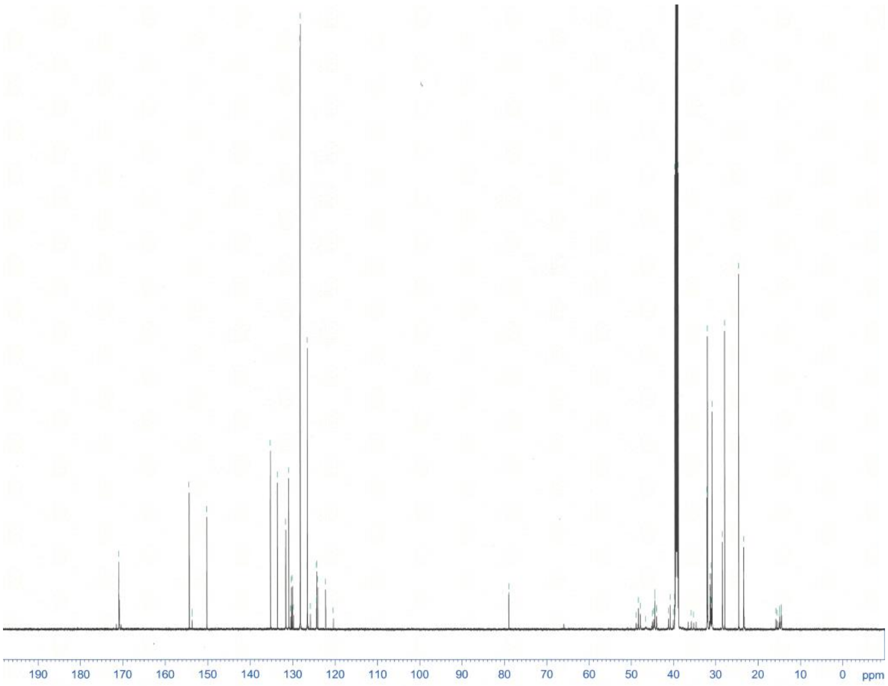

13C-NMR (151MHz,298.2 K), DMSO-*d6*

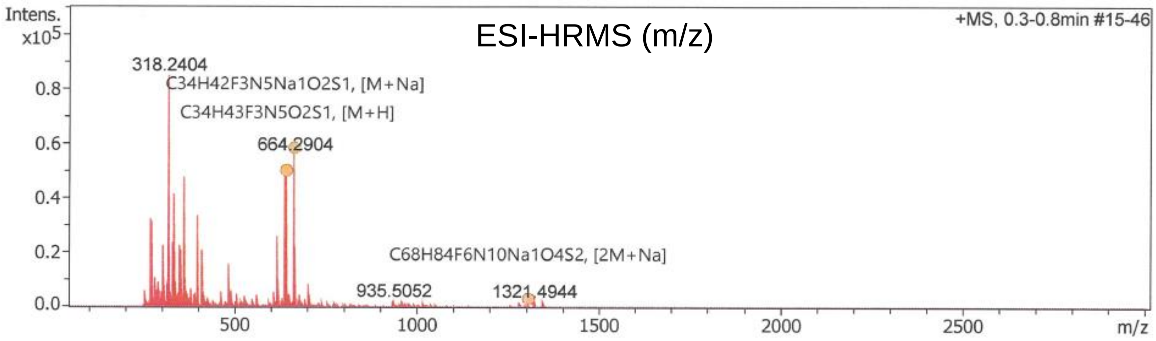

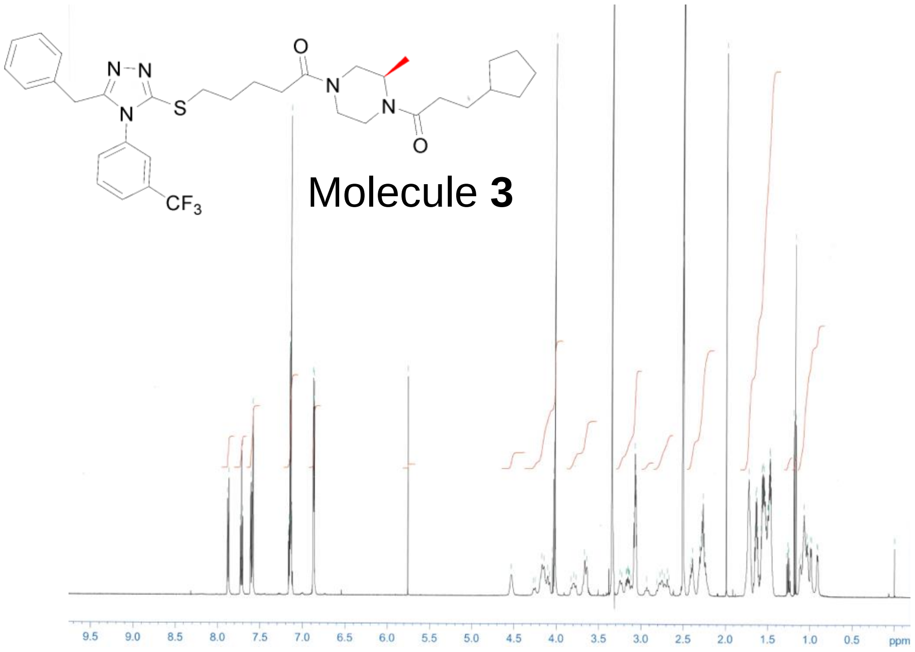

Molecule **3**

1H-NMR (600 MHz, 298.2 K), DMSO-*d6*

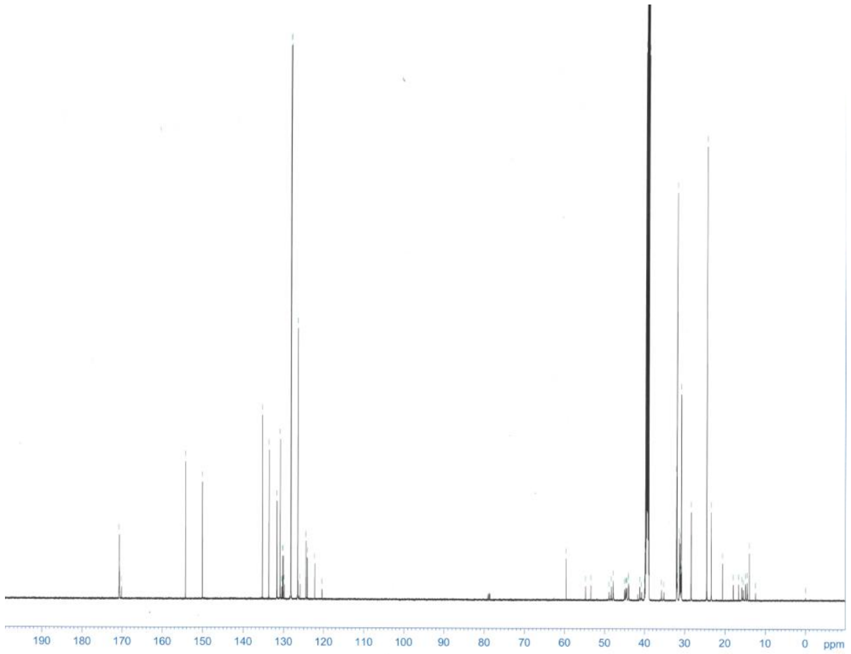

13C-NMR (151MHz,298.2 K), DMSO-*d6*

ESI-HRMS (m/z)

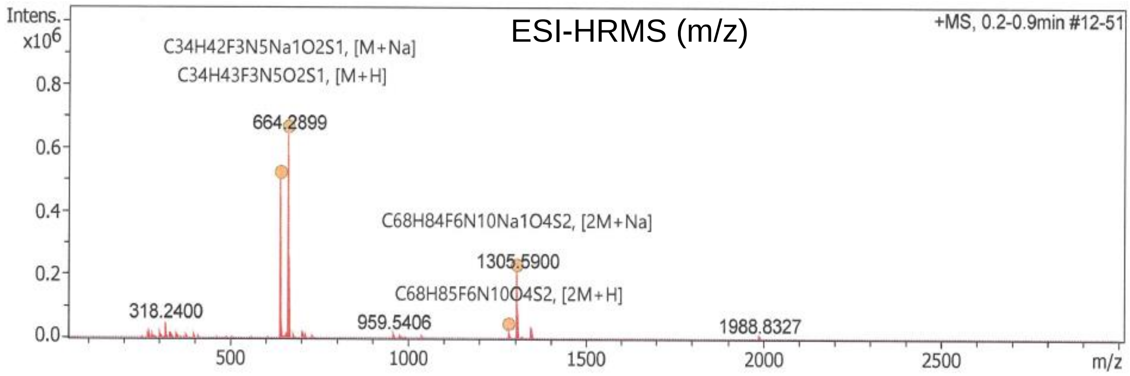

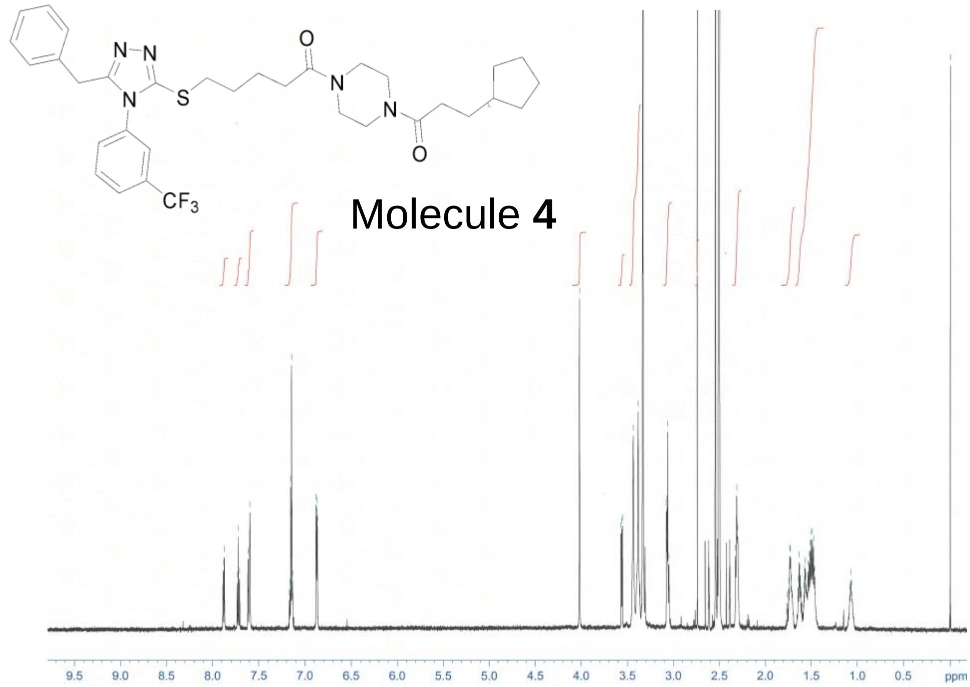

Molecule **4**

1H-NMR (600 MHz, 298.2 K), DMSO-*d6*

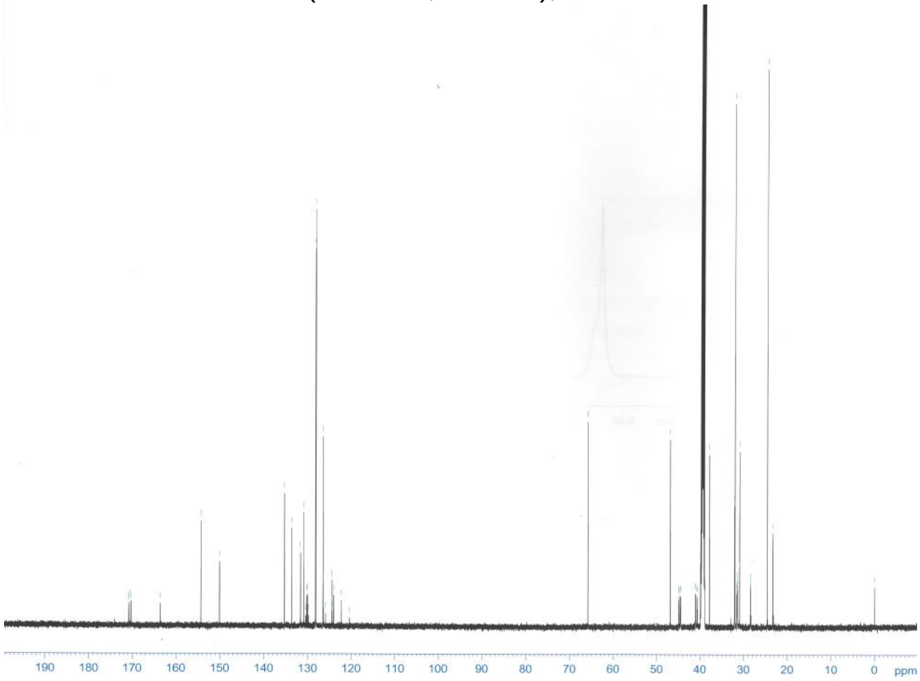

13C-NMR (151MHz,298.2 K), DMSO-*d6*

ESI-HRMS (m/z)

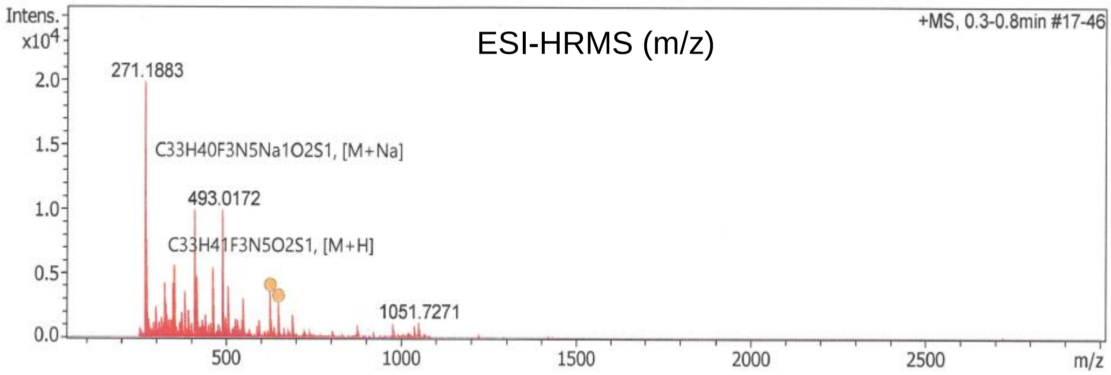

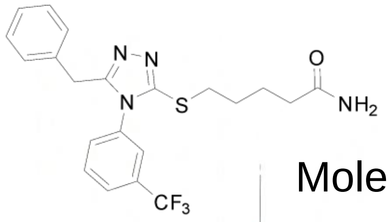

Molecule **5**

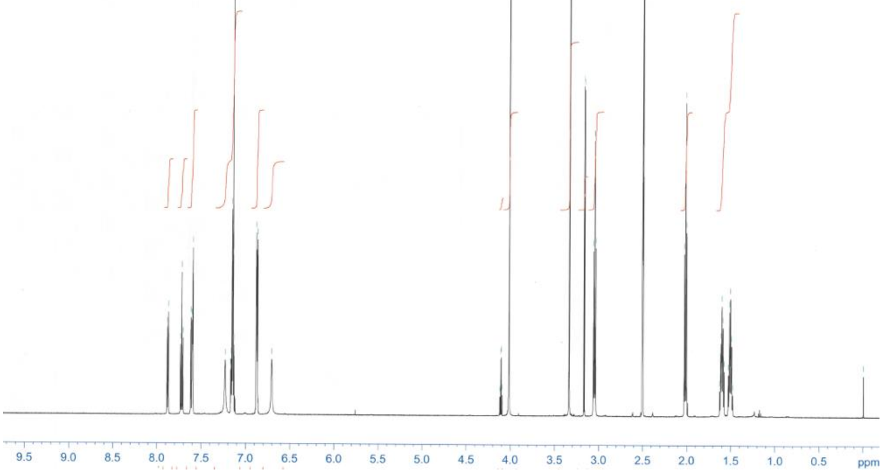

1H-NMR (600 MHz, 298.2 K), DMSO-*d6*

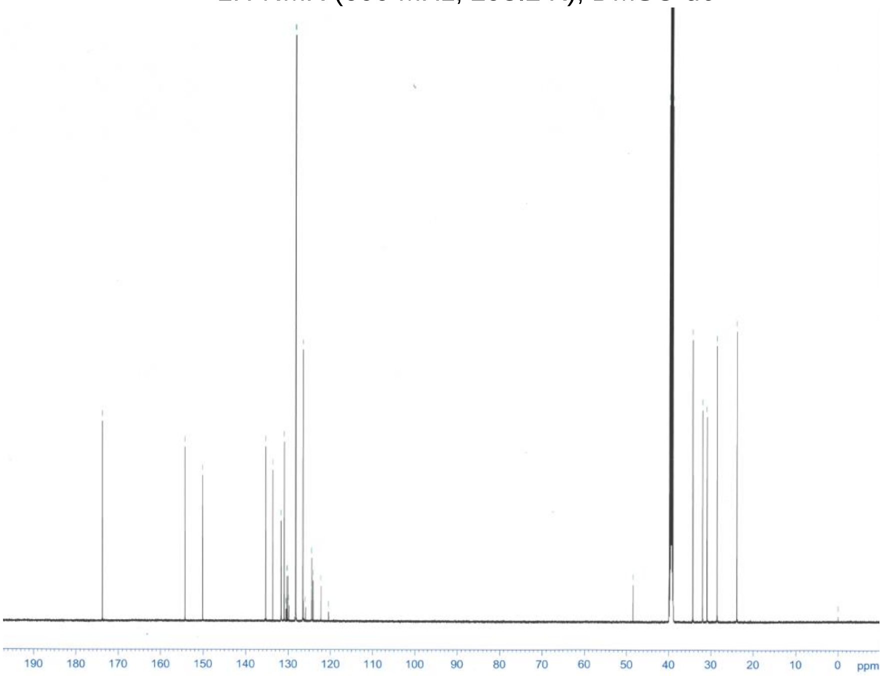

13C-NMR (151MHz,298.2 K), DMSO-*d6*

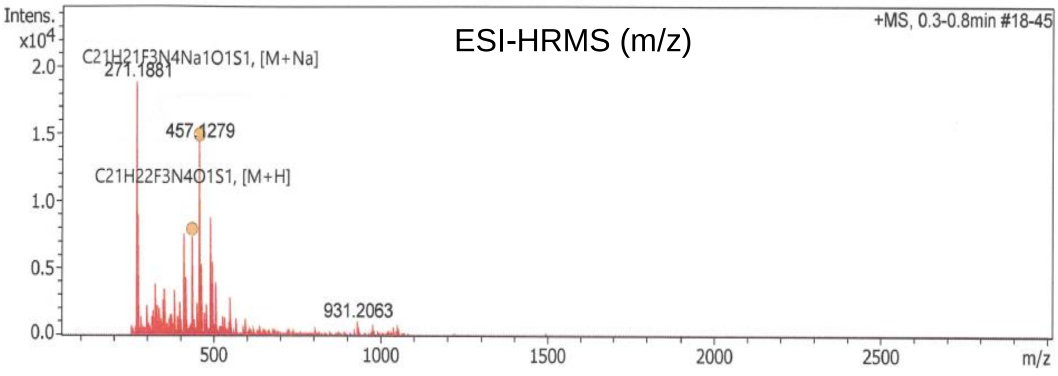

ESI-HRMS (m/z)

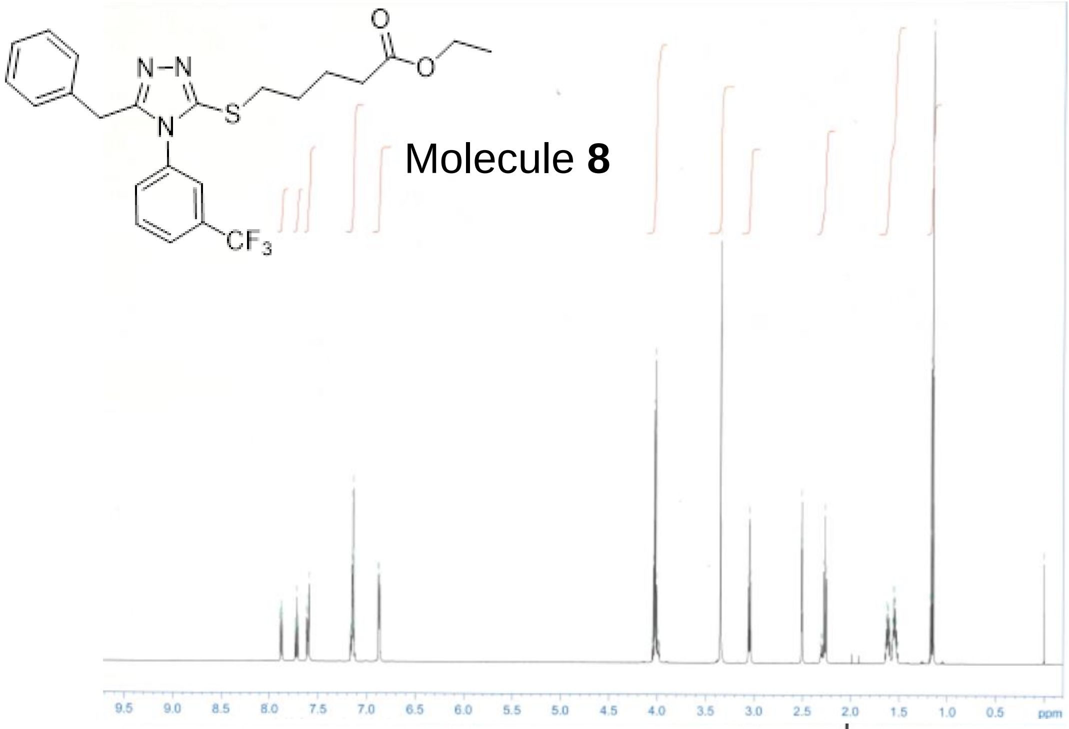

Molecule **8**

1H-NMR (600 MHz, 298.2 K), DMSO-*d6*

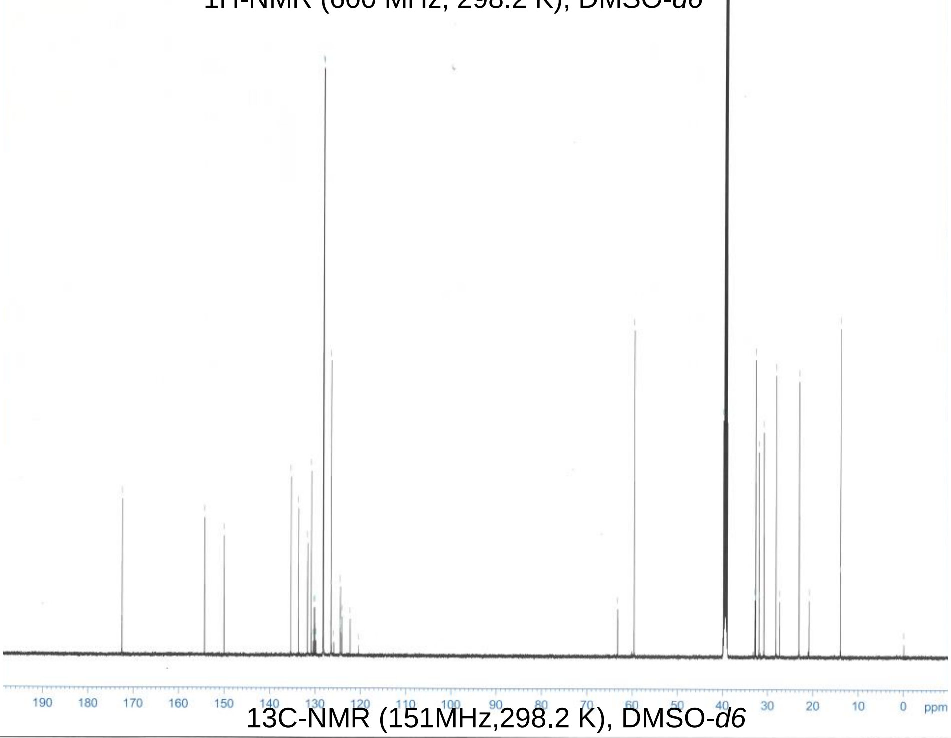

13C-NMR (151MHz,298.2 K), DMSO-*d6*

ESI-HRMS (m/z)

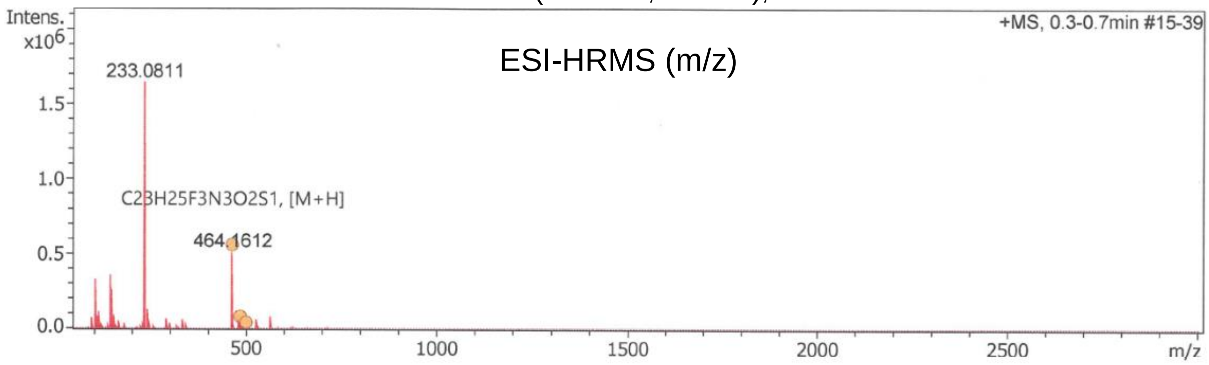

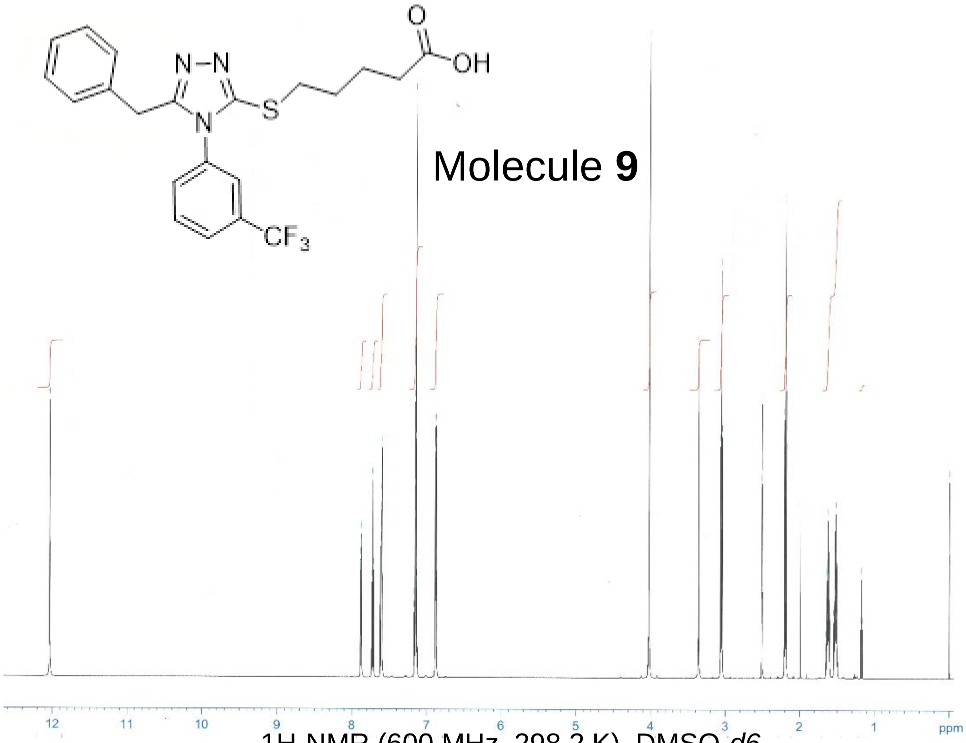

Molecule **9**

1H-NMR (600 MHz, 298.2 K), DMSO-*d6*

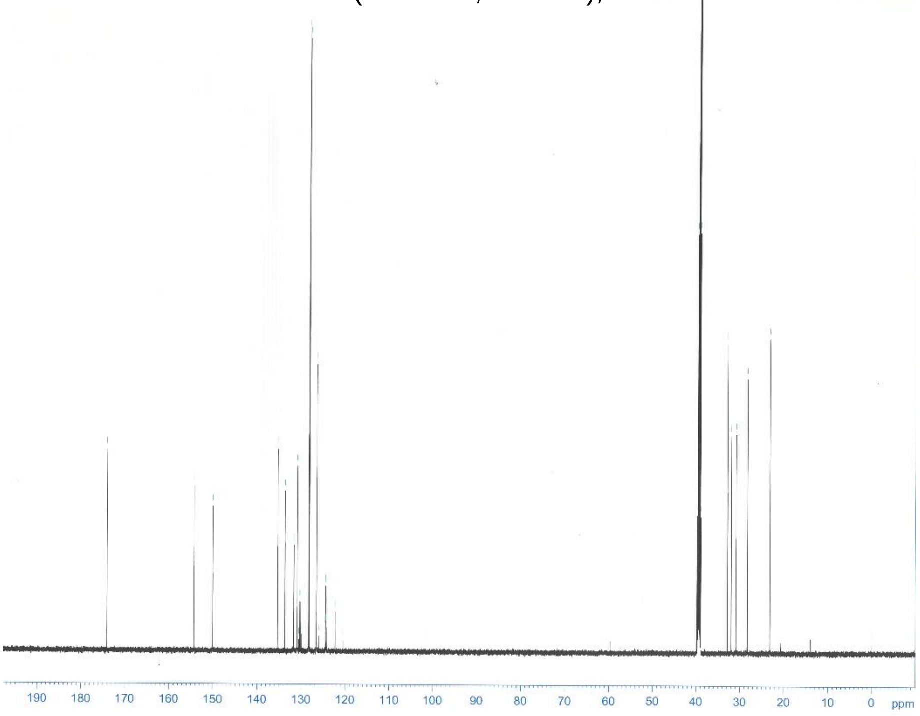

13C-NMR (151MHz,298.2 K), DMSO-*d6*

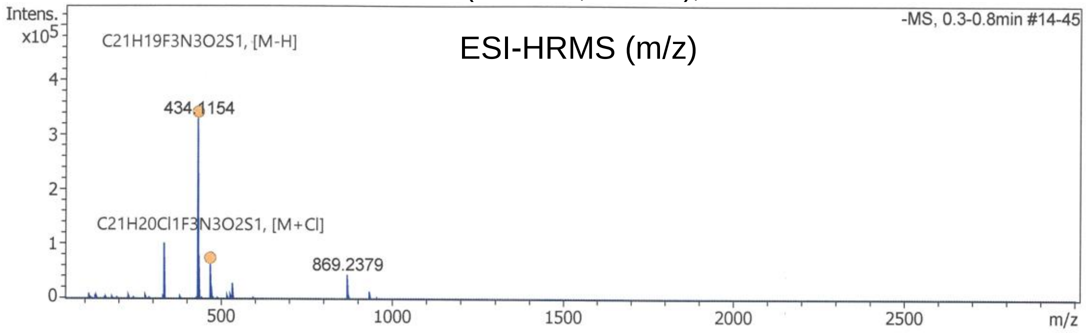

ESI-HRMS (m/z)

Probe **6**

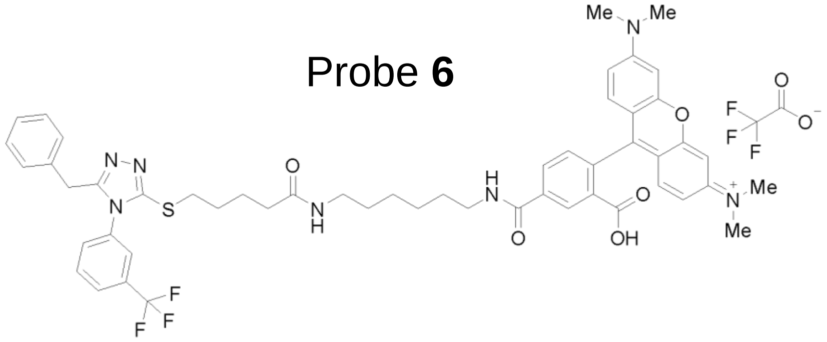

ESI-HRMS (m/z)

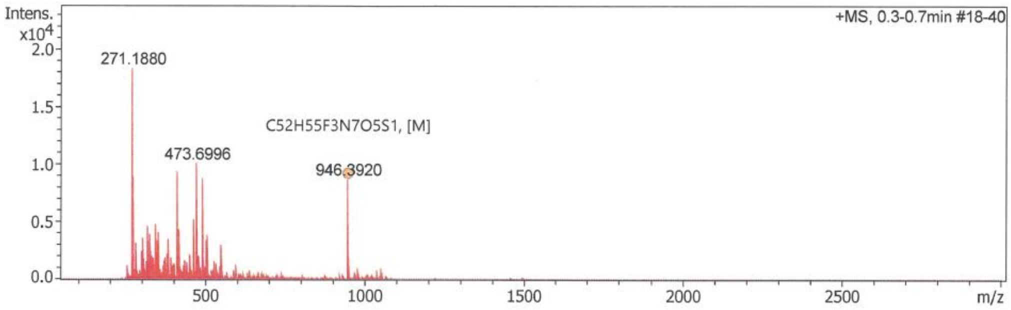

References

(1) S. A. F. Rostom, M. H. Badr, H. A. A. E. Razik and H. M. A. Ashour, *Eur J Med Chem,* 2017, **139**, 263–279.
(2) Y. Kuang, M. J. C. Long, J. Zhou, J. Shi, Y. Gao, C. Xu, L. Hedstrom and B. Xu, *J Biol Chem*, 2014, **289** (42), 29208–29218.
(3) F. Meitinger, M. Ohta, K. Y. Lee, S. Watanabe, R. L. Davis, J. V. Anzola, R. Kabeche, D. A. Jenkins, A. K. Shiau, A. Desai and K. Oegema, *Nature*, 2020, **585** (7825), 440–446.
(4) A. Perron, Y. Nishikawa, J. Iwata, H. Shimojo, J. Takaya, K. Kobayashi, I. Imayoshi, N. M. Mbenza, M. Takenoya, R. Kageyama, Y. Kodama and M. Uesugi, *J Biol Chem*, 2018, **293** (21), 8285–8294.
(5) S. Plimpton, *J Comput Phys*, 1995, **117** (1), 1–19.
(6) S. L. Mayo, B. D. Olafson and W. A. Goddard, *J Phys Chem*, 1990, **94** (26), 8897–8909.
(7) K. Momma and F. Izumi, *J Appl Crystallogr*, 2011, **44** (6), 1272–1276.
